# Supplementary material for: Sen1 Is Recruited to Replication Forks via Ctf4 and Mrc1 and Promotes Genome Stability
Source: Cell Rep. 2020 Feb 18;30(7):2094–2105.e9. doi: 10.1016/j.celrep.2020.01.087 (PMC7034062; doi:10.1016/j.celrep.2020.01.087)
Supplement: Document S1. Figures S1–S5 [file mmc1.pdf]

Cell Reports, Volume *30*

Supplemental Information

Sen1 Is Recruited to Replication Forks via Ctf4 and Mrc1 and Promotes Genome Stability

Rowin Appanah, Emma Claire Lones, Umberto Aiello, Domenico Libri, and Giacomo De Piccoli

A)

Protein ID	MW (kDa)	TAGGED (peptide N)	UNTAGGED (peptide N)
CTF4	104	365	0
MCM4	115	123	0
POL2	256	97	0
SEN1	253	96	0
TOP1	90	85	0
DPB3	59	83	17
MCM3	108	61	0
SLD5	40	34	3
MCM6	113	34	0
DPB2	61	27	6
MRC1	124	25	0
MCM5	86	24	0
HHF1	11	22	0
TOF1	141	22	0

G)

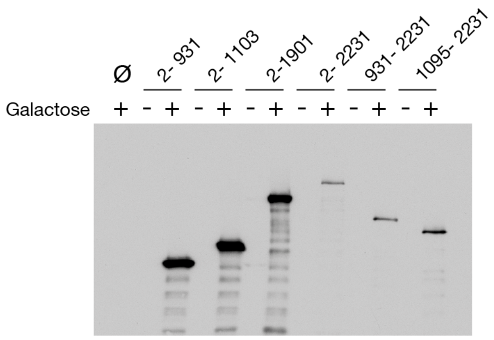

B)

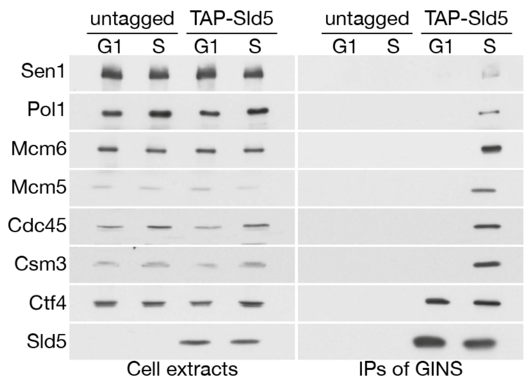

H)

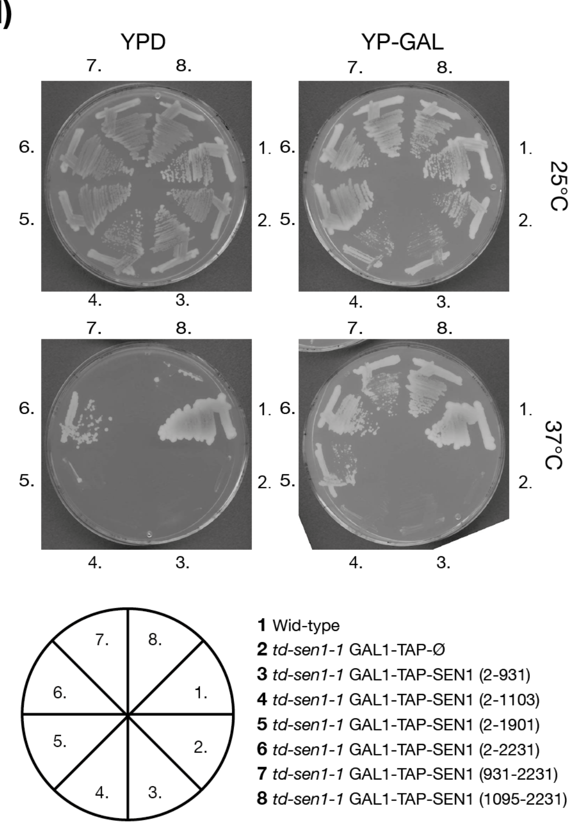

C)

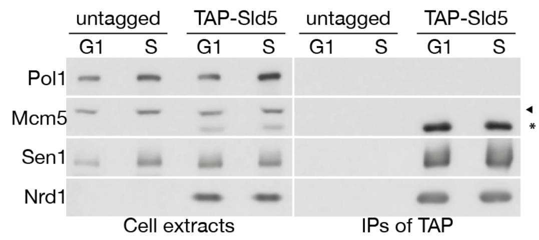

D)

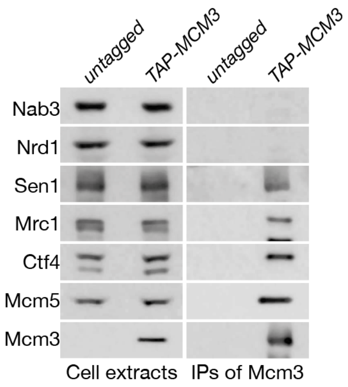

I)

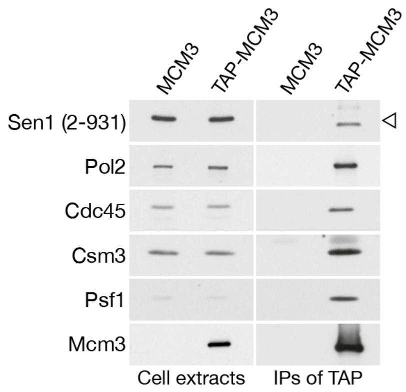

E)

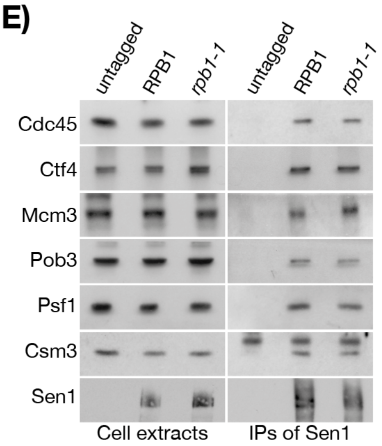

F)

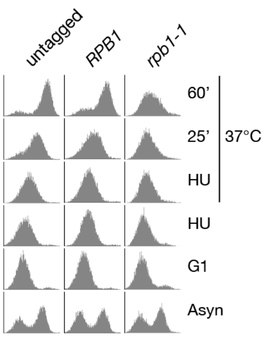

J)

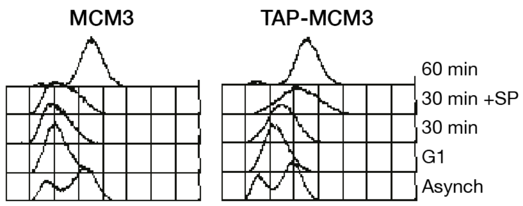

Figure S1. The replisome binds Sen1 in S phase (related to Fig 1)**. A)** Example of the mass spectrometry analysis obtained from the double purification of Sld5 and Mcm4. **B)** Cells carrying the *SEN1-9MYC* allele with a *SLD5* or *TAP-SLD5* allele were synchronously released from G1 into S phase for 30 min at 24°C. Cell extracts were incubated with anti-TAP beads and analysed by immunoblotting. **C)** Nrd1 does not interact with the replisome. *NRD1* or *NRD1-TAP* cells were released from G1 arrest into S phase for 30 min at 24°C. Cell extracts were incubated with anti-TAP beads and analysed by immunoblotting. **D)** Mcm3 immunoprecipitates Sen1 but neither Nrd1 nor Nab3. *MCM3* or *TAP-MCM3* cells were arrested in G1 and released into S phase for 30 min at 24˚C. Cell extracts were incubated with anti-TAP beads and analysed by immunoblotting. **E)** Sen1 interacts *in vivo* with the replisome independently of RNAPII transcription. Wild type or *rpb1-1* cells, either carrying an untagged or TAP-tagged allele of *SEN1*, were arrested in G1 and released in medium containing 0.2 M HU for 75 min at 24˚C. Cultures were then shifted to 37˚C for 1 h. Inactivation of *rpb1-1* cells at 37˚C for 1 h has been shown to lead to a substantial loss of Rpb1-1 from chromatin (Zanton and Pugh, 2006; Kim *et al.*, 2010), to a loss of elongation factors Spt5 and Spt16 (Tardiff, Abruzzi and Rosbash, 2007), to a loss of Sen1 recruitment at highly transcribed genes (Alzu *et al.*, 2012) and to the termination of transcription (Nonet *et al.*, 1987). Cells were then released for 25 min at 37˚C in fresh medium so to allow the synthesis of the bulk of the DNA. Cell extracts were incubated with anti-TAP beads and analysed by immunoblotting. **F)** FACS analysis of the experiment in **E)**. **G)** Cells carrying several different N-terminally tagged truncations of *SEN1* under the *GAL1* promoter were grown to exponential phase in YPRAF, divided in two cultures and transferred to either fresh YPRAF or to YPGAL for 2 h. Protein extracts were analysed by immunoblotting with an anti-HA antibody. **H)** (Top) Cells carrying a temperature-sensitive allele *td-sen1-1* and different fragments of *SEN1* under the *GAL1-3HA* promoter were plated, according to the schematic presented, on YPD or YPGAL and incubated at either 24°C or 37°C. (Bottom) schematic of the plated strains. **I)** The interaction of Sen1 (2-931) with TAP-Mcm3 during S phase is specific. Experiments carrying an untagged or a TAP-tagged allele of *MCM3* were conducted as in Fig 1C. **J)** FACS samples from the experiment are shown.

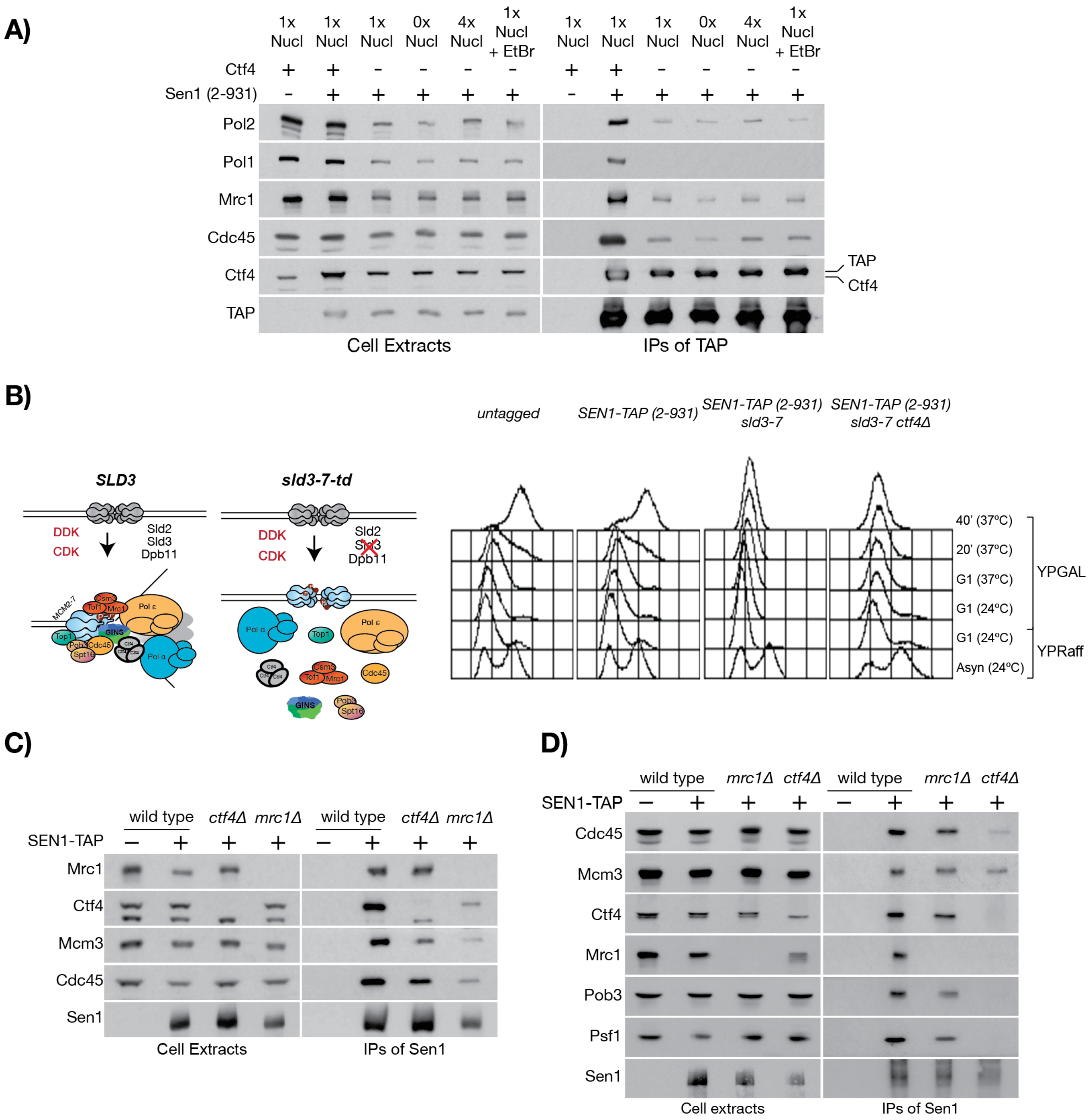
A)
1x Nucl
0x Nucl
4x Nucl
1x Nucl + EtBr
Ctf4
Sen1 (2-931)
Pol2
Pol1
Mrc1
Cdc45
Ctf4
TAP
TAP
Ctf4
Cell Extracts
IPs of TAP
B)
SLD3
sld3-7-td
DDK
CDK
Sld2
Sld3
Dpb11
Pol ε
Pol α
Top1
Cdc45
GINS
Mrc1
Tof1
Csm3
Pob3
Spt16
untagged
SEN1-TAP (2-931)
SEN1-TAP (2-931) sld3-7
SEN1-TAP (2-931) sld3-7 ctf4Δ
40' (37ºC)
20' (37ºC)
G1 (37ºC)
G1 (24ºC)
G1 (24ºC)
Asyn (24ºC)
YPGAL
YPRaff
C)
wild type
ctf4Δ
mrc1Δ
SEN1-TAP
Mrc1
Ctf4
Mcm3
Cdc45
Sen1
Cell Extracts
IPs of Sen1
D)
wild type
mrc1Δ
ctf4Δ
SEN1-TAP
Cdc45
Mcm3
Ctf4
Mrc1
Pob3
Psf1
Sen1
Cell extracts
IPs of Sen1

Figure S2. **Mrc1 and Ctf4 mediate Sen1 binding to the replisome** (related to Fig 2). **A)** Cells carrying the *GAL1-TAP-SEN1 (2- 931)* construct or the empty control were grown in YPGAL, arrested in G1 phase using α-factor and released in S phase for 20 min at 30°C. The samples were then used for IPs using TAP beads and treated with the indicated amount of nuclease or ethidium bromide (50 µg/ml). Ctf4 and TAP-Sen1 (2-931) have similar sizes and run closely in gel electrophoresis. **B)** (Left) Schematic representation of the system used in the experiment shown in Fig 2D; (Right) FACS profile of the experiment conducted. Cells were grown in YPRAF at 24°C, arrested in G1 and either harvested, or resuspended in YPGAL at 24°C for 35 min to induce the expression of Sen1 (2-931) and Ubr1, shifted to 37°C for 1 h to inactivate/degrade td-Sld3-7 and then released in S phase for 20 min at 37°C. **C)** Wild type, *ctf4*Δ and *mrc1*Δ cells, carrying a TAP-tagged or untagged allele of *SEN1*, were arrested in G1 and synchronously released in S phase for 30 min at 24˚C. Cell extracts were incubated with anti-TAP beads and the immunoprecipitated material was analysed by immunoblotting. **D)** Immunoblotting analysis of cell extracts and IP material from anti-TAP beads. The experiment was conducted an in **C)**, except cultures were incubated with formaldehyde before collection.

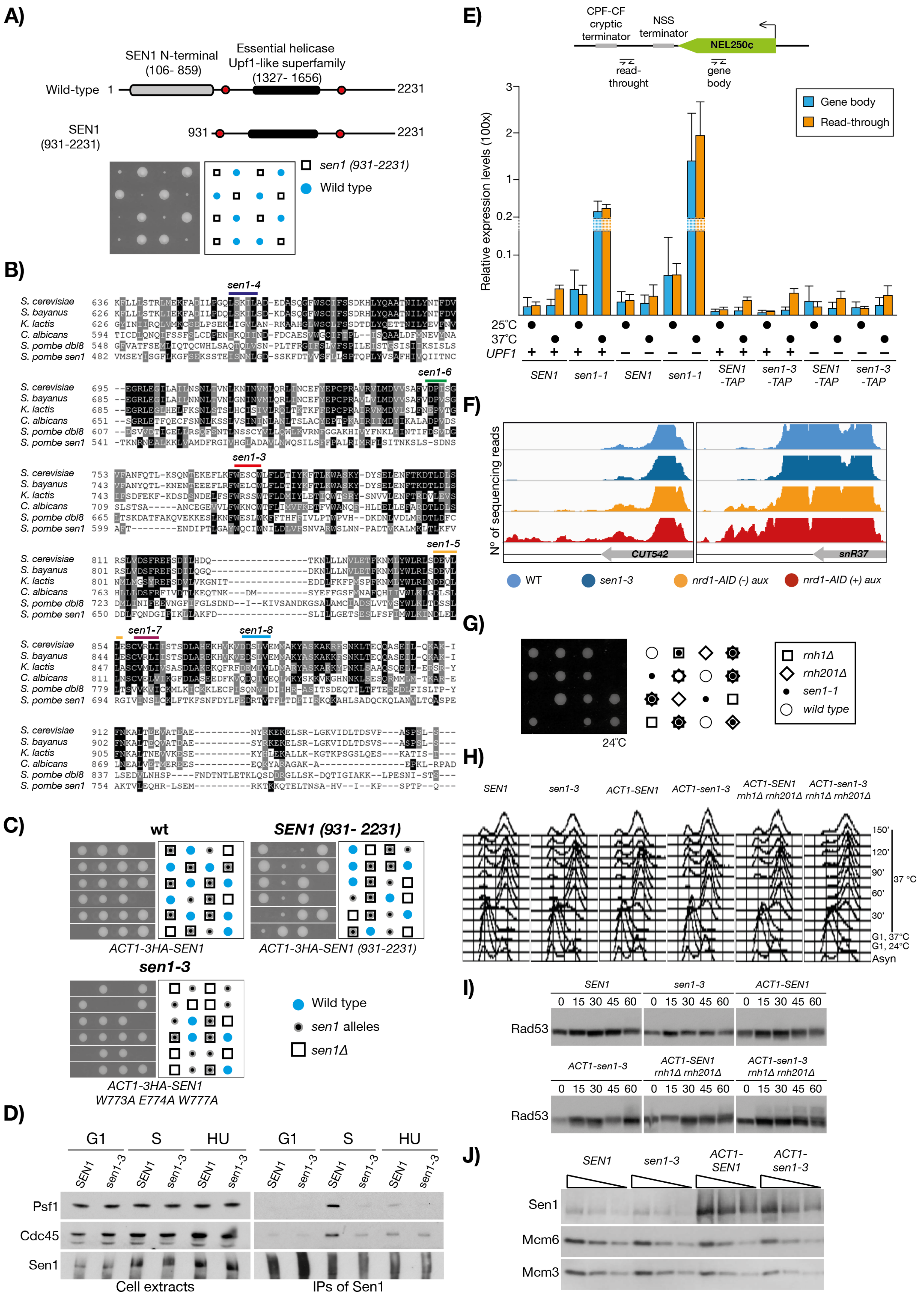

A)
SEN1 N-terminal (106- 859)
Essential helicase Upf1-like superfamily (1327- 1656)
Wild-type 1
2231
SEN1 (931-2231)
931
2231
sen1 (931-2231)
Wild type
B)
sen1-4
sen1-6
sen1-3
sen1-5
sen1-7
sen1-8
S. cerevisiae
S. bayanus
K. lactis
C. albicans
S. pombe dbl8
S. pombe sen1
C)
wt
SEN1 (931- 2231)
ACT1-3HA-SEN1
ACT1-3HA-SEN1 (931-2231)
sen1-3
ACT1-3HA-SEN1 W773A E774A W777A
Wild type
sen1 alleles
sen1Δ
D)
G1
S
HU
SEN1
sen1-3
Psf1
Cdc45
Sen1
Cell extracts
IPs of Sen1
E)
CPF-CF cryptic terminator
NSS terminator
NEL250c
read-throught
gene body
Relative expression levels (100x)
Gene body
Read-through
25˚C
37˚C
UPF1
SEN1
sen1-1
SEN1 -TAP
sen1-3 -TAP
F)
N° of sequencing reads
CUT542
snR37
WT
sen1-3
nrd1-AID (-) aux
nrd1-AID (+) aux
G)
24˚C
rnh1Δ
rnh201Δ
sen1-1
wild type
H)
SEN1
sen1-3
ACT1-SEN1
ACT1-sen1-3
ACT1-SEN1 rnh1Δ rnh201Δ
ACT1-sen1-3 rnh1Δ rnh201Δ
150'
120'
90'
60'
30'
37 °C
G1, 37°C
G1, 24°C
Asyn
I)
SEN1
sen1-3
ACT1-SEN1
0 15 30 45 60
Rad53
ACT1-sen1-3
ACT1-SEN1 rnh1Δ rnh201Δ
ACT1-sen1-3 rnh1Δ rnh201Δ
J)
SEN1
sen1-3
ACT1-SEN1
ACT1-sen1-3
Sen1
Mcm6
Mcm3

Figure S3. The N-terminal of Sen1 is important for cell growth and is conserved in yeasts (related to Fig 3 and 4)**. A)** Tetrad analysis of a diploid yeast strain carrying the *SEN1/sen1 (1-931Δ)* alleles. Plates were imaged after 5 days of growth on YPD at 24°C. **B)** Alignment of *Saccharomyces cerevisiae* Sen1 domain interacting with the replisome (636-931) with its orthologues from *Saccharomyces bayanus*, *Kluyveromyces lactis*, *Candida albicans* and *Schizosaccharomyces pombe dbl8* and *sen1*. The mutations used in the screen were selected to mutate conserved amino acids predicted to be on the surface (www.predictprotein.org). **C)** Analysis of the *ACT1-3HA-SEN1* mutants. Tetrad analyses were conducted on diploids yeast strains carrying *SEN1/sen1*Δ, and ectopically integrated *ACT1-3HA-SEN1* alleles at the *leu2-3,112* locus. Plates were imaged after 3 days of growth on YPDA at 24°C. **D)** Sen1-3 show greatly reduced interaction with RFs. Wild type and *sen1-3* cells were arrested in G1 and synchronously released for 30 min in fresh medium (S) or for 90 min in medium containing 0.2 M HU. At the indicated times, cultures were treated with formaldehyde before being collected. The cross-linked cell extracts and the immunoprecipitated material from anti-TAP beads were analysed by immunoblotting**. E)** (Top) Schematic representation of the gene analysed and the probes used to assess defects in transcription termination; (Bottom) RT-qPCR analysis of RNAs derived from the indicated strains. *NEL025c* is a non-coding region as described in (Wyers *et al.*, 2005). Cells were grown to exponential phase and incubated for 3 h at the indicated temperature before being collected. The signal is presented as the expression level relative to the housekeeping gene *ACT1* (triplicate biological repeats). **F)** Snapshots illustrating RNA Pol II density detected by CRAC on two NNS complex targets (one CUT and one snoRNA) in the indicated strains. An *nrd1-AID* strain grown in the presence or absence of auxin is included as a control for transcription termination (dataset from (Candelli *et al.*, 2018)). **G)** *sen1-1* is lethal in the absence of *RNH201* and *RNH1*. Examples are shown of tetrad analyses conducted from yeast diploids strains with the *SEN1/sen1-1 RNH1/rnh1*Δ and *RNH201/rnh201*Δ genotype. Plates were imaged after 4 days of growth on YPDA at 24°C. **H)** FACS analysis of the cell cycle progression in cells *SEN1*, *sen1-3, ACT1-SEN1, ACT1-sen1-3, ACT1-SEN1 rnh1*Δ *rnh201*Δ and *ACT1-sen1-3 rnh1*Δ *rnh201*Δ. Cells were grown to the exponential phase at 24°C, arrested in G1, shifted to 37°C for 1 hour in G1, and released in S phase at 37°C. The samples were collected at the indicated time points. **I)** *ACT1-sen1-3 rnh1*Δ *rnh201*Δ cells show activation of Rad53 during DNA replication at 37°C. Western blot analysis of samples taken from the experiment shown in **H). J)** Analysis of the protein levels of the alleles *SEN1*, *sen1-3*, *ACT1-SEN1* and *ACT1-sen1-3*, all carrying a 3HA tag, and loading controls Mcm3 and Mcm6.

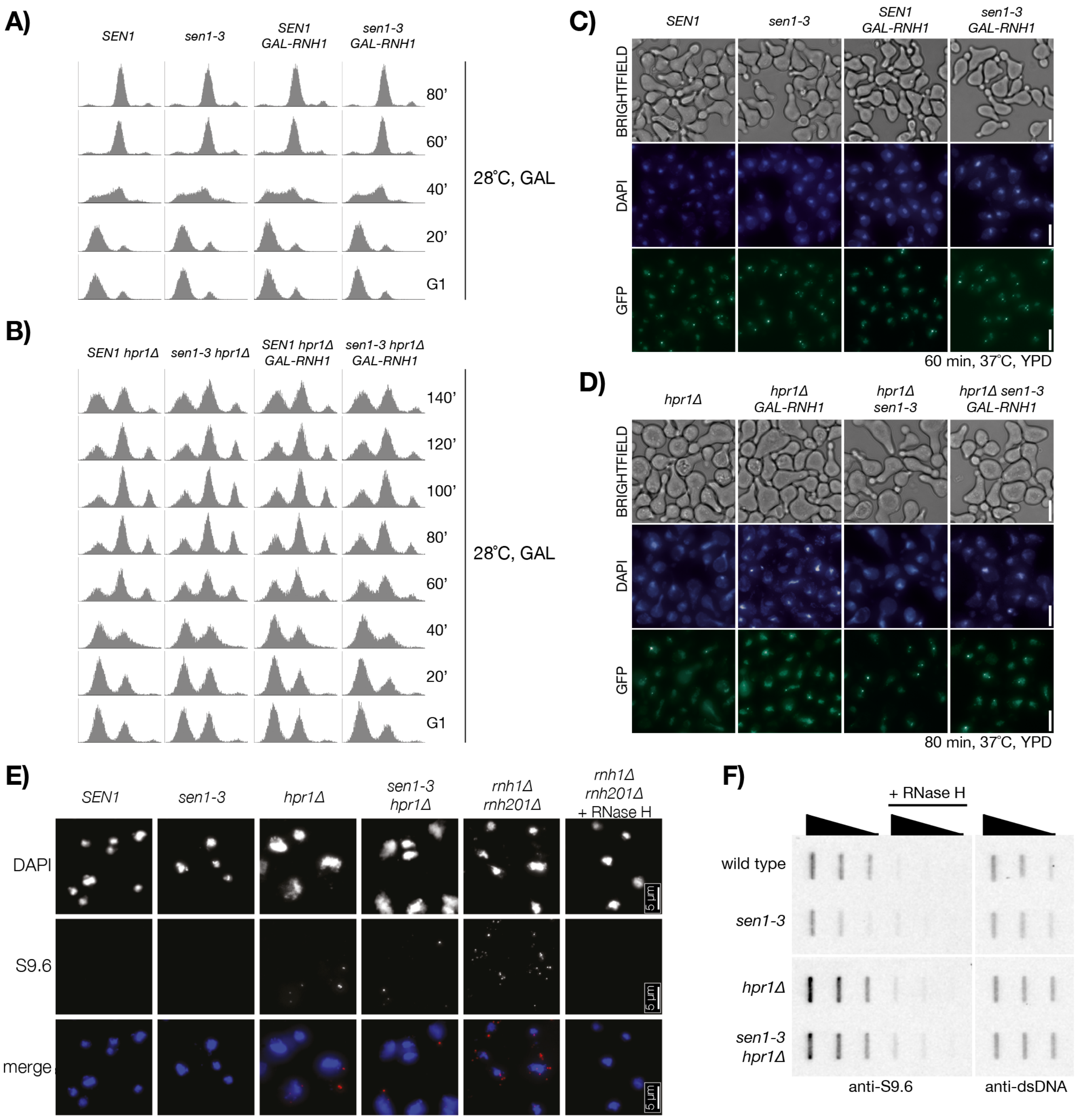

A)
SEN1
sen1-3
SEN1 GAL-RNH1
sen1-3 GAL-RNH1
80'
60'
40'
20'
G1
28°C, GAL
B)
SEN1 hpr1Δ
sen1-3 hpr1Δ
SEN1 hpr1Δ GAL-RNH1
sen1-3 hpr1Δ GAL-RNH1
140'
120'
100'
80'
60'
40'
20'
G1
28°C, GAL
C)
SEN1
sen1-3
SEN1 GAL-RNH1
sen1-3 GAL-RNH1
BRIGHTFIELD
DAPI
GFP
60 min, 37°C, YPD
D)
hpr1Δ
hpr1Δ GAL-RNH1
hpr1Δ sen1-3
hpr1Δ sen1-3 GAL-RNH1
BRIGHTFIELD
DAPI
GFP
80 min, 37°C, YPD
E)
SEN1
sen1-3
hpr1Δ
sen1-3 hpr1Δ
rnh1Δ rnh201Δ
rnh1Δ rnh201Δ + RNase H
DAPI
S9.6
merge
5 µm
F)
+ RNase H
wild type
sen1-3
hpr1Δ
sen1-3 hpr1Δ
anti-S9.6
anti-dsDNA

Figure S4. Analysis of the recombination and DNA:RNA hybrids in *hpr1*Δ and *hpr1*Δ *sen1-3* (related to Fig 4**)**. **A-D)** FACS analysis of the DNA replication dynamics and examples of the microscopy data of the experiments shown in Fig 4F are shown. Cells were grown to exponential phase in YPRAF at 28°C, arrested in G1, resuspended in YPGAL for 1 h and synchronously released in S phase in YPGAL (triplicate biological repeats). Scale bar = 5 µm **E)** Examples of the immunohistochemistry analysis of DNA:RNA shown in Fig 4G (triplicate biological repeats). Scale bar = 5 µm **F)** Analysis of R-loops *ex vivo***.** Cells were grown to exponential phase, arrested in G1 and then synchronously released in S phase for 30 min at 24°C. DNA:RNA hybrids double-stranded DNA were recovered in nuclease-free water and 1, 0.5 and 0.25 µg/µl dilutions of nucleic acid samples were prepared. The samples were then either treated with a commercially-sourced RNase H (or mock-treated), transferred onto nylon membrane and probed against using either the S9.6 antibody (that recognise R-loops) or an anti-dsDNA antibody.

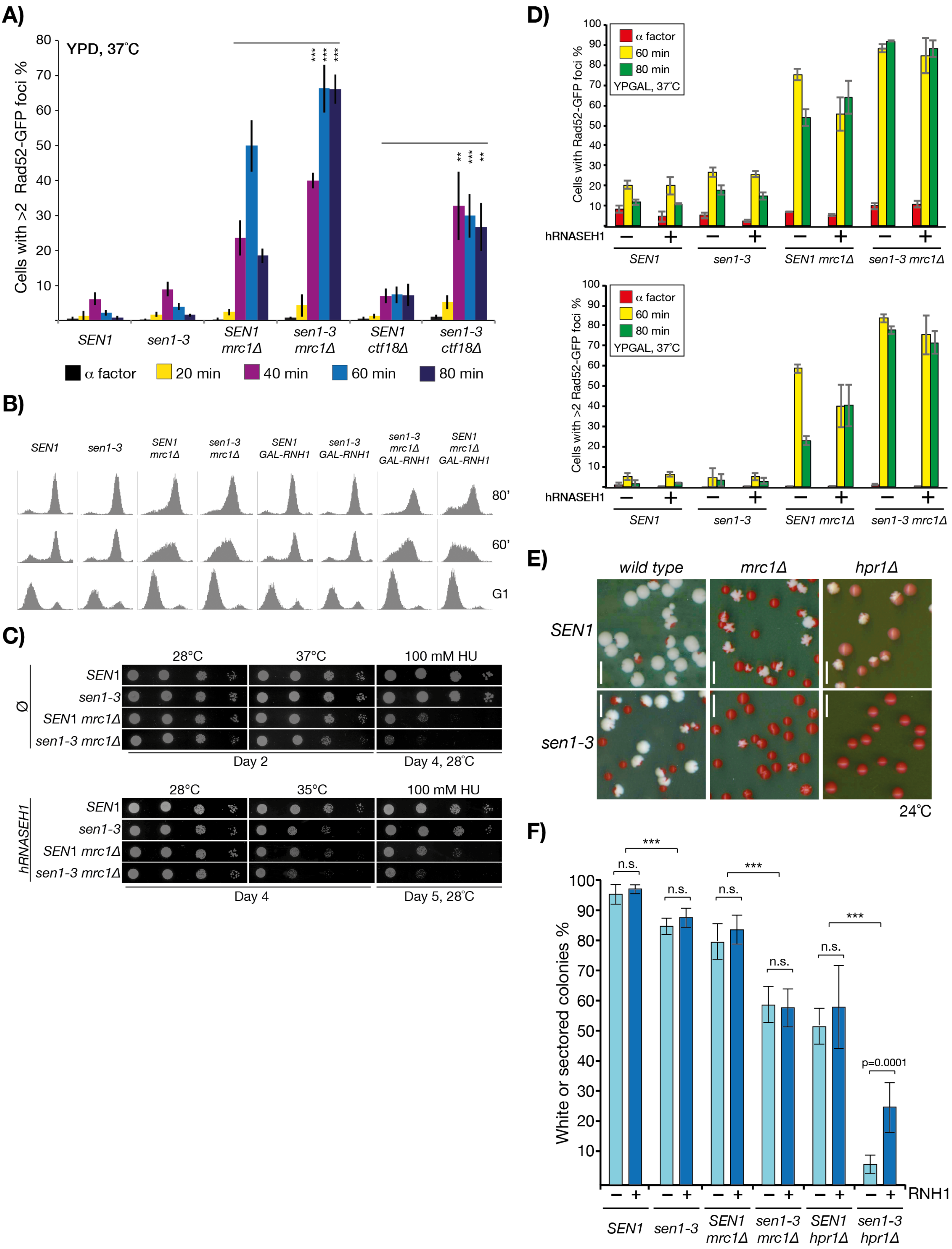
A)
YPD, 37˚C
Cells with >2 Rad52-GFP foci %
SEN1
sen1-3
SEN1 mrc1Δ
sen1-3 mrc1Δ
SEN1 ctf18Δ
sen1-3 ctf18Δ
α factor
20 min
40 min
60 min
80 min
B)
SEN1
sen1-3
SEN1 mrc1Δ
sen1-3 mrc1Δ
SEN1 GAL-RNH1
sen1-3 GAL-RNH1
sen1-3 mrc1Δ GAL-RNH1
SEN1 mrc1Δ GAL-RNH1
80'
60'
G1
C)
28°C
37°C
100 mM HU
Ø
SEN1
sen1-3
SEN1 mrc1Δ
sen1-3 mrc1Δ
Day 2
Day 4, 28˚C
28°C
35°C
100 mM HU
hRNASEH1
Day 4
Day 5, 28˚C
D)
Cells with >2 Rad52-GFP foci %
α factor
60 min
80 min
YPGAL, 37˚C
hRNASEH1
SEN1
sen1-3
SEN1 mrc1Δ
sen1-3 mrc1Δ
E)
wild type
mrc1Δ
hpr1Δ
SEN1
sen1-3
24˚C
F)
White or sectored colonies %
n.s.
***
p=0.0001
RNH1
SEN1
sen1-3
SEN1 mrc1Δ
sen1-3 mrc1Δ
SEN1 hpr1Δ
sen1-3 hpr1Δ

Figure S5. Overexpression of *RNH1* does not suppress the defects in *mrc1*Δ *sen1-3* (related to Fig 5**)**. **A)** Samples from experiments show in Fig 5D were scored for the presence of two or more foci per cell. ** p<0.05, *** p<0.01). **B)** FACS analysis of the experiment shown in Fig 5G. **C)** *hRNASEH1* overexpression does not suppress the defects observed in *sen1-3 mrc1*Δ cells. *hRNASEH1* was overexpressed from a 2 micron multicopy plasmid under the strong GPD^{TDH3} promoter. *SEN1*, *sen1-3*, *mrc1*Δ and *mrc1*Δ *sen1-3* cells were transformed with an empty or GPD^{TDH3}-*hRNASEH1* plasmid. Eight independent clones were pooled together and used for dilution spotting in medium lacking histidine, so to maintain the selective pressure for the plasmid. The cells carrying *GDP-hRNH1* grew more slowly and scans of their growth were taken at later times (Bottom panel). Serial dilution spotting (1:10) of the indicated strains is shown. **D)** *hRNASEH1* overexpression does not suppress the increase in recombination in *sen1-3 mrc1*Δ. Cell cultures were grown overnight at 24°C in medium lacking histidine to the exponential phase. Cells were diluted, resuspended in YPD, and left to grow for the length of one cell cycle. Cells were arrested in G1, shifted to 37°C for 1 h still with α-factor, and released in S phase at 37°C. Cells were taken at the indicated times, fixed, and analysed (triplicate biological repeats). **E)** Examples of the plasmid loss phenotype observed in the strains shown in Fig 5I (plasmid with 1 origin). Scale bar: 5 mm. **F)** The indicated strains, either carrying the *GAL1-RNH1* construct integrated at *leu2-3,112* or not, were transformed with the pRS315-*ADE2* plasmid. The experiment was performed as in Fig 5I, except that all media used contained galactose. n.s. = not significant, *** p<0.001.
