# Supplementary material for: Sen1 Is Recruited to Replication Forks via Ctf4 and Mrc1 and Promotes Genome Stability
Source: Cell Rep. 2020 Feb 18;30(7):2094–2105.e9. doi: 10.1016/j.celrep.2020.01.087 (PMC7034062; doi:10.1016/j.celrep.2020.01.087)
Supplement: Document S2. Article plus Supplemental Information [file mmc3.pdf]

Report

Cell Reports

Sen1 Is Recruited to Replication Forks via Ctf4 and Mrc1 and Promotes Genome Stability

Graphical Abstract

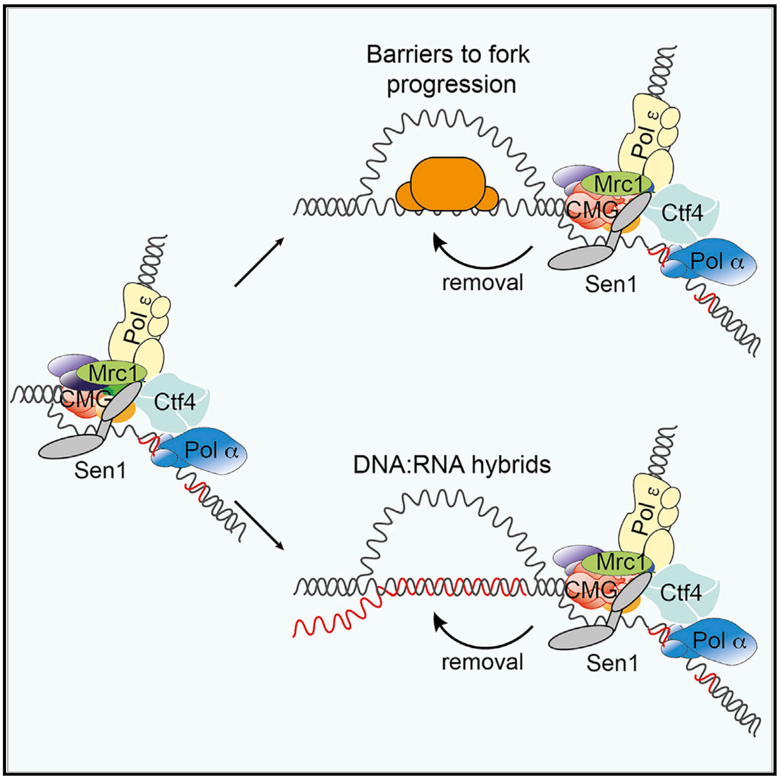

Highlights

- The N-terminal domain of Sen1 mediates replisome association
- Ctf4 and Mrc1 bind to Sen1 and promote its recruitment to the replisome
- The *sen1-3* allele abrogates replisome binding, but not transcription termination
- *sen1-3* cells are sensitive to high levels of R-loops and defects of S phase checkpoint

Authors

Rowin Appanah, Emma Claire Lones, Umberto Aiello, Domenico Libri, Giacomo De Piccoli

Correspondence

g.de-piccoli@warwick.ac.uk

In Brief

Appanah et al. identify the transcription termination helicase Sen1 as a bona fide component of the replisome. Sen1 binds the replisome via its N-terminal domain and Ctf4 and Mrc1. The allele *sen1-3* breaks this interaction without affecting transcription termination. *sen1-3* cells show sensitivity to R-loops levels and increased genomic instability.

Appanah et al., 2020, Cell Reports *30*, 2094–2105
February 18, 2020 © 2020 The Authors.
https://doi.org/10.1016/j.celrep.2020.01.087

CellPress

OPEN ACCESS
CellPress

Cell Reports
Report

Sen1 Is Recruited to Replication Forks via Ctf4 and Mrc1 and Promotes Genome Stability

Rowin Appanah,[1] Emma Claire Lones,[1] Umberto Aiello,[2] Domenico Libri,[2] and Giacomo De Piccoli[1,3,*]
[1]Warwick Medical School, University of Warwick, CV4 7AL Coventry, UK
[2]Institut Jacques Monod, CNRS, UMR7592, Université Paris Diderot, Paris Sorbonne Cité, Paris, France
[3]Lead Contact
*Correspondence: g.de-piccoli@warwick.ac.uk
https://doi.org/10.1016/j.celrep.2020.01.087

SUMMARY

DNA replication and RNA transcription compete for the same substrate during S phase. Cells have evolved several mechanisms to minimize such conflicts. Here, we identify the mechanism by which the transcription termination helicase Sen1 associates with replisomes. We show that the N terminus of Sen1 is both sufficient and necessary for replisome association and that it binds to the replisome via the components Ctf4 and Mrc1. We generated a separation of function mutant, *sen1-3*, which abolishes replisome binding without affecting transcription termination. We observe that the *sen1-3* mutants show increased genome instability and recombination levels. Moreover, *sen1-3* is synthetically defective with mutations in genes involved in RNA metabolism and the S phase checkpoint. *RNH1* overexpression suppresses defects in the former, but not the latter. These findings illustrate how Sen1 plays a key function at replication forks during DNA replication to promote fork progression and chromosome stability.

INTRODUCTION

The maintenance of genome stability requires the complete and faithful duplication of DNA in every cell cycle. Yet several obstacles impede the progression of replication forks (RFs), and these must be removed to avoid stalling and increased chromosome instability. A significant barrier to RF progression is transcription. First identified in bacteria, collisions between RFs and transcription bubbles also represent a major obstacle for DNA synthesis in eukaryotes, leading to defects in chromosome maintenance and an increase in levels of recombination (Liu and Alberts, 1995; Helmrich et al., 2011, 2013; Prado and Aguilera, 2005; Kim et al., 2010; Hamperl et al., 2017; Tran et al., 2017). In order to complete the full duplication of the chromosomes, replisomes must therefore overcome transcriptional barriers, removing both the DNA-bound RNA polymerase subunits and any DNA:RNA hybrids formed during transcription. These hybrids, usually limited to eight base pairs, occur naturally during RNA transcription and are typically removed when the RNA polymerase is disengaged from the DNA (Aguilera and García-Muse, 2012; Westover et al., 2004).

At specific chromosomal loci, extended DNA:RNA hybrids can also form behind the site of RNA synthesis, through the re-annealing of nascent RNA to the template DNA and the displacement of the non-template DNA. These structures, named R-loops, form preferentially at highly transcribed genes with a high GC skew and can extend up to 1 kb in higher eukaryotes (Aguilera and García-Muse, 2012; Skourti-Stathaki et al., 2014). Formation of R-loops is favored by head-on collisions between RFs and actively transcribing complexes (Hamperl et al., 2017; Lang et al., 2017), and their non-physiological accumulation, coupled to chromatin modification, is deleterious for genome stability (García-Pichardo et al., 2017). Several pathways minimize the formation and stability of R-loops. For instance, the promotion of transcription processivity (Hazelbaker et al., 2013), transcription termination (Kim et al., 2004; Luke et al., 2008), timely processing, export or degradation of nascent mRNA (Huertas and Aguilera, 2003; Pfeiffer et al., 2013), or preventing torsional stress that arises during transcription (El Hage et al., 2010, 2014) all minimize R-loops' levels. Nevertheless, once formed, R-loops must be removed. A key role in R-loop removal is fulfilled by the RNase H enzymes that specifically digest RNA molecules within DNA:RNA hybrids (Cerritelli and Crouch, 2009). In addition, several helicases can unwind DNA:RNA hybrids *in vitro*, including Sgs1 (Chang et al., 2017) and Pif1 (Boulé and Zakian, 2007). One such helicase, Sen1, is believed to play an essential role in the removal of R-loops from the DNA in yeast (Mischo et al., 2011).

Sen1 is an Upf1-like helicase that plays a key role in transcription termination (Jankowsky, 2011; Steinmetz et al., 2006; Ursic et al., 1997; Porrua and Libri, 2013). Sen1 binds to the free 5′ ends of either RNA or DNA substrates and unwind both double-stranded DNA (dsDNA) and DNA:RNA hybrids (Han et al., 2017; Leonaitė et al., 2017; Martin-Tumasz and Brow, 2015; Porrua and Libri, 2013). *In vitro* analysis shows that Sen1 has high activity but limited processivity on DNA:RNA hybrid substrates (Han et al., 2017). Mechanistically, when Sen1 engages with nascent RNA exiting from a stalled RNA polymerase II (RNAPII), the helicase seemingly exerts a force on the polymerase to "push" it, either overcoming the stalling of RNAPII or disengaging it from the template DNA (Porrua and Libri, 2013; Han et al., 2017). *In vivo* data also suggest that Sen1 is capable of removing RNAPII from the DNA it is bound to, thus terminating transcription (Steinmetz et al., 2006; Schaughency et al., 2014; Hazelbaker et al., 2013). In fact, a mutation in the catalytic domain of Sen1 (*sen1-1*) confers defects in transcription termination at non-permissive temperatures, leading to extensive readthrough

 © 2020 The Authors.
This is an open access article under the CC BY license (http://creativecommons.org/licenses/by/4.0/).

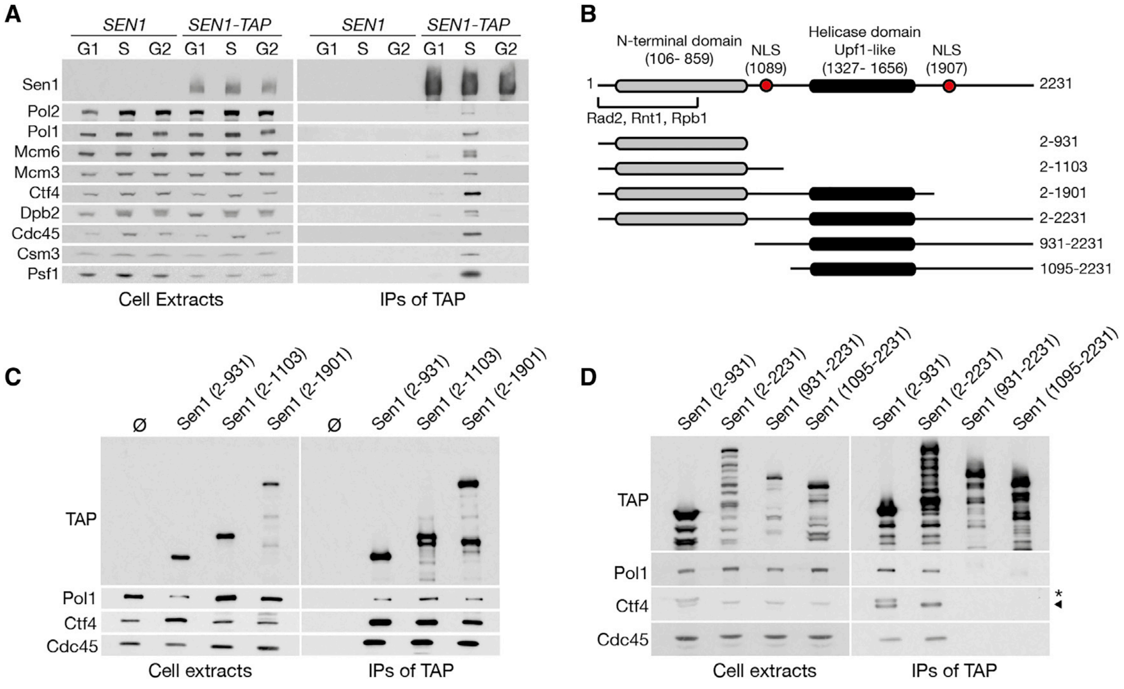

Figure 1. Sen1 Interacts with the Replisome during S Phase through Its N-Terminal Domain

(A) *SEN1* or *SEN1-TAP* cells were arrested in G1, harvested immediately, or released for either 30 min (S phase) or 60 min (G2 phase). Cell extracts and IP material were analyzed by immunoblotting (IB).

(B) Schematic of Sen1 constructs used.

(C) TAP-tagged fragments of Sen1, IPed from cells in S phase, were analyzed by IB.

(D) TAP-tagged fragments of Sen1 were analysed as above, except 4× cells were used for the IP of the fragments containing the last 330 C-terminal amino acids.

of several transcription units (Steinmetz et al., 2006), accumulation of R-loops, and increased recombination (Mischo et al., 2011). Because of these defects, the viability of *sen1-1* cells depends on several repair factors (Mischo et al., 2011; Alzu et al., 2012). Moreover, depletion of Sen1 leads to slow DNA replication and the accumulation of abnormal structures on 2D gels (Alzu et al., 2012; Brambati et al., 2018).

Given its relatively low abundance and processivity (Mischo et al., 2018; Han et al., 2017), Sen1 needs to be recruited at, or close to, sites where it can enact its biological function. Sen1 is recruited to the termination sites of cryptic-unstable transcripts (CUTs) and small nucleolar RNAs (snoRNAs) by binding to Nab3 and Nrd1, which both dock onto nascent RNA (Arigo et al., 2006; Porrua et al., 2012; Creamer et al., 2011). Nrd1 also interacts with Rpo21^{Rpb1} (the largest subunit of RNAPII) early in the transcription cycle (Vasiljeva et al., 2008), thus restricting Sen1-dependent termination to short transcription units (Gudipati et al., 2008). Sen1 also promotes termination of some genes downstream of the polyadenylation site, acting with Rat1 (Mischo et al., 2011; Rondón et al., 2009), possibly by directly binding Rpo21 via its N-terminal domain (Chinchilla et al., 2012). Finally, it is likely that Sen1 is recruited at other genomic sites in a transcription-independent fashion. The human ortholog of Sen1 (Senataxin) co-localizes with 53BP1 to sites of DNA damage in a checkpoint-dependent manner (Yüce and West, 2013). Moreover, in *S. cerevisiae*, Sen1 co-localizes with replisome components and sites of bromodeoxyuridine (BrdU) incorporation (Alzu et al., 2012). However, the mechanism through which Sen1 is recruited at RFs has yet to be described. The significance of recruiting Sen1 to RFs is also poorly understood, as it has been impossible thus far to determine whether the defects in DNA replication upon inactivation of Sen1 are an indirect consequence of deregulated transcription termination, of a failure in R-loop removal, or the direct result of an important function of Sen1 at RFs. Here, we show that Sen1 binds the replisome during S phase through its N-terminal domain, map its binding site, generate a mutant that breaks this interaction, and explore the consequences of the loss of the helicase from RFs on chromosome stability.

RESULTS

Sen1 Interacts with the Replisome via Its N-Terminal Domain

The replisome is a complex and dynamic machine that relies on multiple interactions between its constituent proteins (Bell and Labib, 2016; Burgers and Kunkel, 2017). As part of a mass spectrometry (MS) screen to identify factors transiently or weakly associated to the core replisome, we observed that Sen1 co-purifies with the CMG helicase in *S. cerevisiae* (Figure S1A). To verify the MS data, we immunoprecipitated (IPed) Sen1 from extracts of yeast cells synchronized in G1, S, and G2. We observed that Sen1 interacted with replisome components only in S phase (Figure 1A). Immunoprecipitation (IP) of the GINS component

OPEN
ACCESS
CellPress

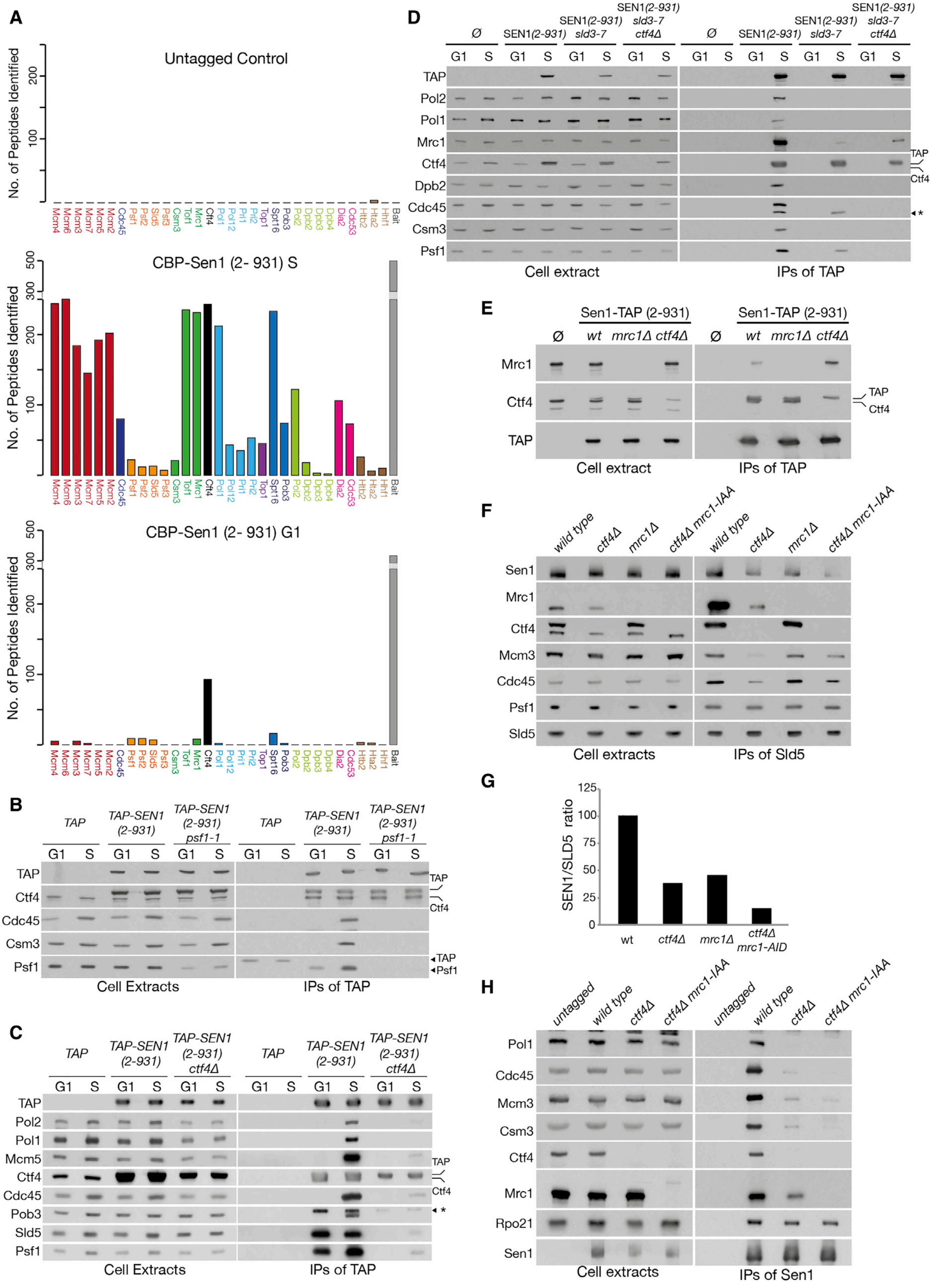

(legend on next page)

Sld5 corroborated this observation (Figure S1B). Sen1 interacts with replisomes independently of either Nrd1 or Nab3 (Figures S1C and S1D) and independently of ongoing transcription (Figures S1E and S1F), as previously observed (Alzu et al., 2012). To further explore this interaction and its biological function, we mapped the interaction sites both in the replisome and Sen1.

Sen1 contains an extended N-terminal domain and an essential and conserved helicase domain (Leonaitė et al., 2017). To identify a region of Sen1 that is sufficient for binding replisomes, we generated TAP-tagged constructs of Sen1, expressed under an inducible *GAL1* promoter (Figure 1B). All fragments containing the helicase domain folded correctly and rescued *sen1-1* lethality at non-permissive temperatures, despite constructs containing the last 330 amino acids of the protein being highly labile (Figures S1G and S1H). We then assessed the ability of the various fragments to interact with the replisome and observed that the N-terminal domain (residues 2–931) of Sen1 was both sufficient and necessary for association with replisomes (Figures 1C and 1D). Similarly, Sen1 (2–931) co-precipitated specifically with replisomes isolated from S phase cells by IP of Mcm3 (a subunit of the CMG helicase) (Figures S1I and S1J). Thus, Sen1 (2–931) contains an interaction site for replisome components.

Sen1 Binding to the Replisome Depends on Ctf4 and Mrc1

To identify specific proteins to which Sen1 binds within the replisome, we compared the G1 and S phase interactome of Sen1 (2–931) via MS analysis. As expected, Sen1 (2–931) IPed with replisomes in S phase (Figure 2A). Interestingly, Ctf4 and GINS co-purified with the bait in G1 as well. This was confirmed by immunoblotting (Figures 2B and 2C). Because Ctf4 and GINS interacts throughout the cell cycle (Gambus et al., 2009), we next analyzed whether Sen1 binds preferentially to one of the components. The interaction between Ctf4 and Sen1 in G1 was unaffected by inactivating GINS via the *psf1-1* allele (Figure 2B; Takayama et al., 2003), but GINS no longer IPed with Sen1 (2–931) in G1 in the absence of Ctf4 (Figure 2C). These data indicate that Sen1 (2–931) binds to Ctf4 in the absence of other replisome components.

Interestingly, Sen1 (2–931) retained some affinity to the replisome in the absence of Ctf4 (Figure 2C, right panel), independently of DNA (Figure S2A). This suggests that Sen1 interacts with at least another subunit of the replisome. To screen for such factors, we analyzed whether any component of the replisome binds to Sen1 (2–931) in cells progressing into S phase in the absence of origin firing. We used *td-sld3-7* cells that cannot initiate chromosome replication at 37°C following inactivation and degradation of td-Sld3-7 (Kamimura et al., 2001; Kanemaki and Labib, 2006; Figure S2B). In control cells, Sen1 (2–931) co-purified with all tested replisome components in S phase (Figure 2D). In *td-sld3-7* cells, Sen1 IPed predominantly with Ctf4 and GINS but also weakly with the replisome component Mrc1. Strikingly, Sen1 (2–931)'s affinity for Mrc1 increased in a td-*sld3-7 ctf4Δ* background. We confirmed this in cells arrested in G1 as well (Figure 2E). These observations suggest that both Ctf4 and Mrc1 are binding partners of Sen1 in the replisome. Deletion of either replisome component leads to a decrease in replisome association to Sen1, even following crosslinking to capture weak interactions (Figures S2C and S2D). Because *ctf4Δ mrc1Δ* cells are inviable (Gambus et al., 2009), we generated a *ctf4Δ mrc1-AID* strain, with the auxin-degron fused to Mrc1 (Nishimura et al., 2009) to allow rapid depletion of the protein. The association of Sen1 with the replisome was greatly reduced, although not entirely abolished, in cells with no Ctf4 and Mrc1 (Figures 2F–2H). These data indicate that, although other accessory binding partners might exist within the replisome, Sen1 mainly binds via Ctf4 and Mrc1.

sen1-3 Fails to Bind the Replisome and Is Sensitive to Increased Levels of DNA:RNA Hybrids

Deletion of the N-terminal domain of Sen1 causes pronounced defects in cell growth (Figure S3A). Thus, to investigate the role of Sen1 at RFs, we sought to generate a separation of function allele that is specifically defective for binding to replisomes. By generating truncations of the N-terminal domain, we identified that Sen1 (410–931) was the fragment with the highest affinity for replisomes although Sen1 (622–931) was the smallest construct still able to bind (Figures 3A–3C). By comparison with yeast orthologs of Sen1 (Figure S3B), we identified conserved residues within this region and targeted them for mutagenesis, creating hemagglutinin (HA)-tagged alleles of *SEN1* that were expressed under the strong *ACT1* promoter in *sen1Δ* cells. All the tested mutations supported cell growth, but one allele, combining mutations W773A E774A W777A (henceforth referred to as *sen1-3*) was uniquely defective for interaction with replisomes (Figures 3D and S3C). Similar results were obtained when the *sen1-3* mutation was

Figure 2. Sen1 Binds the Replisome Components Ctf4 and Mrc1

(A) MS analysis of the proteins co-purifying with Sen1 (2–931) was conducted in S and G1 phases.

(B) IB analysis of the proteins IPed with Sen1 (2–931) and an empty control in strains carrying the *PSF1* or *psf1-1* allele. Cells were arrested in G1, shifted to 37°C for 1 h (G1), and then released into S phase for 20 min at 37°C (S).

(C) Sen1 (2–931) binding of GINS in G1 depends on Ctf4. IB analysis of the proteins IPed with Sen1 (2–931) and an empty control, with or without *CTF4*. Cells were arrested in G1 and released in S phase for 20 min at 30°C. Ctf4 and TAP-Sen1 (2–931) have similar sizes and run closely in gel electrophoresis.

(D) IB analysis of the proteins interacting with TAP-Sen1 (2–931) in the presence or absence of origin firing and *CTF4*. Cells were treated as described in Figure S2B. G1 samples were collected before galactose induction.

(E) Wild-type, *mrc1Δ*, or *ctf4Δ* cells expressing TAP-Sen1 (2–931) were arrested in G1. IB analysis of cell extracts and IPs is shown.

(F) Wild-type, *ctf4Δ*, *mrc1Δ*, and *ctf4Δ mrc1-AID* strains were arrested in G1, treated for 1 h with 0.5 mM auxin indole-3-acetic acid (IAA) final concentration, and released in S phase. IB analysis of cell extracts and IPs is shown.

(G) Quantification of the relative signal of Sen1-9MYC versus the TAP-Sld5 signal, normalized against the wild type.

(H) Experiments were conducted as in (F). Wild-type, *ctf4Δ*, and *ctf4Δ mrc1-AID* strains, carrying an untagged or a *SEN1-TAP* allele, were used. Asterisk indicates a non-specific band.

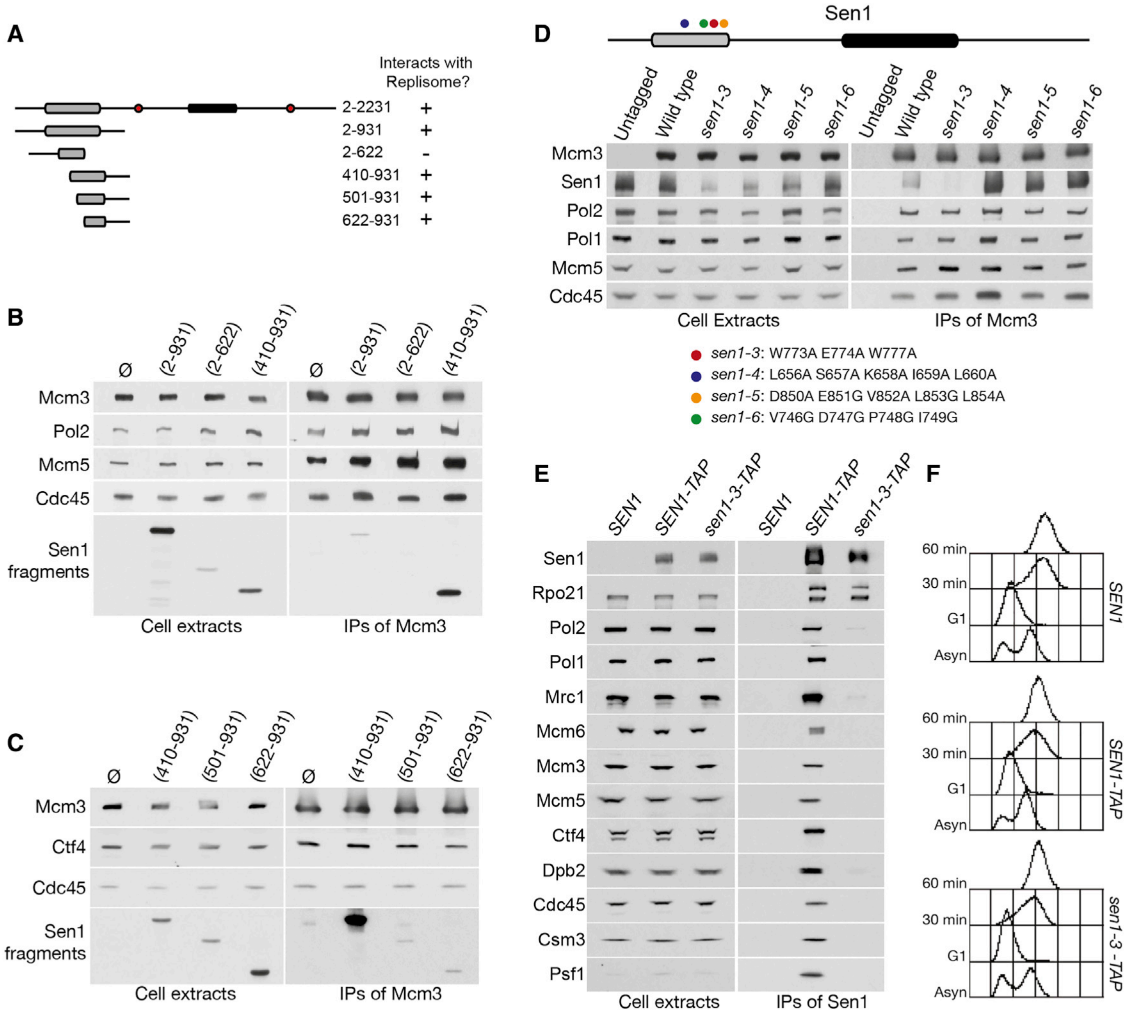

Figure 3. Sen1-3 Does Not Interact with the Replisome

(A) Summary of the ability of N-terminal fragments of Sen1 to interact with the replisome.

(B) Cells carrying different *GAL1-3HA-SEN1* fragments and a *TAP-MCM3* allele were arrested in G1 and released into S phase. The samples were then used for IPs.

(C) Sen1 fragments were analysed as in (B).

(D) Cells carrying *ACT1-3HA-SEN1* wild-type or mutated alleles at an ectopic locus were synchronously released into S phase. IB analysis of cell extracts and IPs is shown.

(E) Cells carrying a *SEN1*, *SEN1-TAP*, or *sen1-3-TAP* allele were arrested in G1 and released into S phase. IB analysis of cell extracts and IPs is shown.

(F) Fluorescence-activated cell sorting (FACS) samples for the experiment in (E).

introduced at the endogenous *SEN1* locus (Figures 3E and 3F), even following crosslinking (Figure S3D). Importantly, Sen1-3 retained wild-type affinity for RNAPII (Rpo21). Hence, *sen1-3* is an allele that abrogates the interaction between Sen1 and replisomes.

Next, we assessed whether the *sen1-3* mutation affects transcription termination, similarly to *sen1-1* cells (Mischo et al., 2011). We assayed the efficiency of termination at two model Nrd1-Nab3-Sen1 target genes, coding for a snoRNA (*SNR13*) and a CUT (*NEL025c*) (Thiebaut et al., 2006; Ursic et al., 1997). Because termination defects lead to longer RNAs that can be targeted by the nonsense-mediated decay, strains lacking *UPF1* were also tested (Culbertson and Leeds, 2003). Cells with the *sen1-3* allele presented no defects in transcription termination, unlike *sen1-1* at 37°C (Figures 4A and S3E). Defects in transcription termination were also analyzed genome-wide by mapping the distribution of RNAPII via the sequencing of nascent RNAs using CRAC (crosslinking and analysis of cDNAs) (Granneman et al., 2009; Candelli et al., 2018). Metagene analysis using a set of validated CUTs (Table S1) shows very similar RNAPII profiles between *SEN1* and *sen1-3* cells, although a clear general termination defect is observed upon depletion of Nrd1 (Figures 4B and S3F). These data indicate that *sen1-3* is proficient in terminating RNAPII transcription.

We then analyzed how the loss of Sen1 from replisomes affects cells. *SEN1* and *sen1-3* cells displayed comparable cell

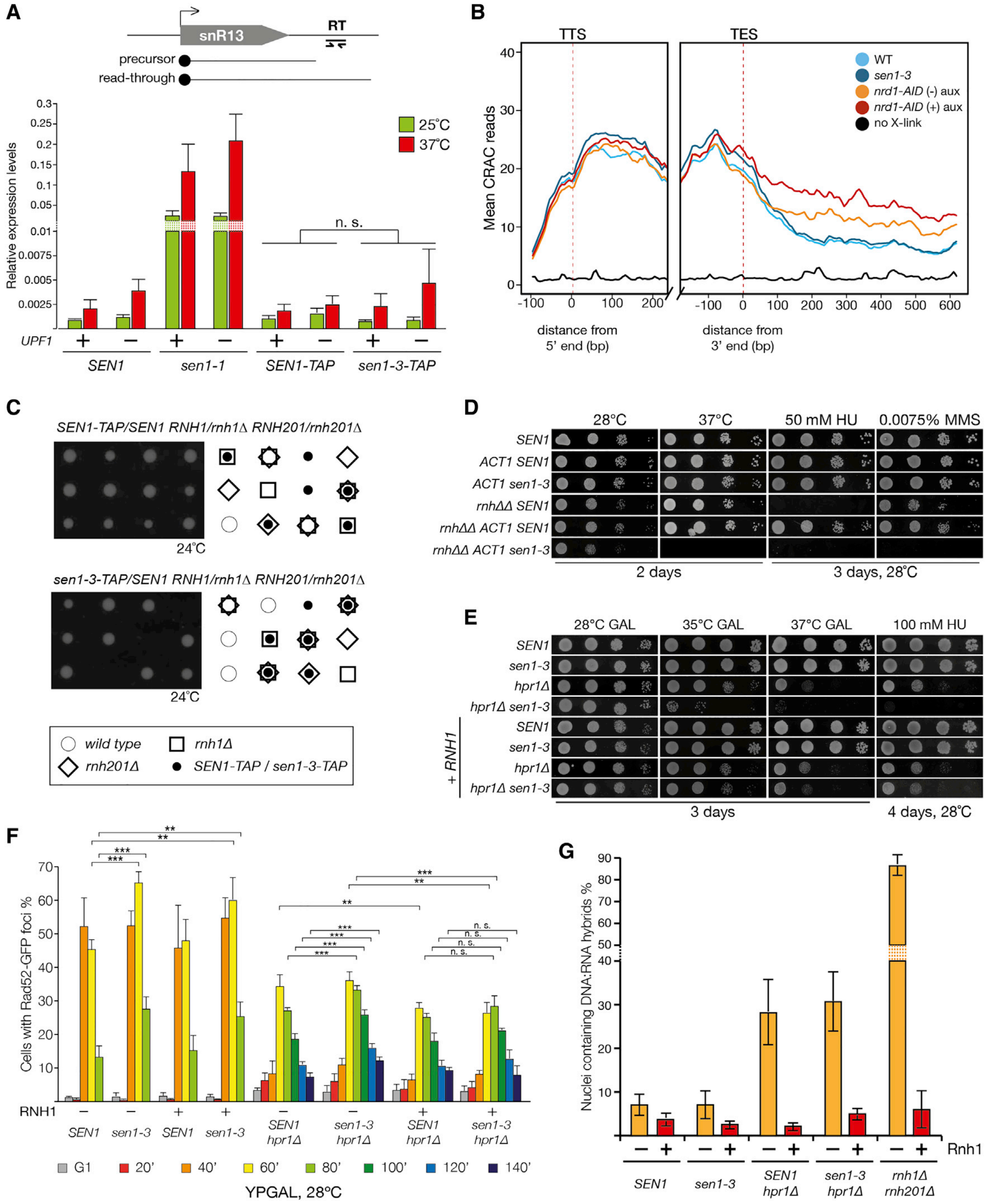

Figure 4. The *sen1-3* Allele Is Proficient in RNAPII Termination but Is Essential in the Absence of RNase H Activity

(A) *sen1-3* cells are proficient for transcription termination. qRT-PCR analysis of RNAs derived from the strains indicated is shown. The ratio of the readthrough fraction (position RT) over the total amount of *SNR13* RNA is shown (triplicate biological repeats). n.s., not significant.

(B) Metagene analysis of RNAPII density detected by CRAC on CUTs. Average read counts are plotted on regions aligned to both the transcription start site (TSS) (left) and the transcript end site (TES) (right) of the CUTs (reads count in Table S1). The profiles of RNAPII density following Nrd1 depletion (*nrd1-AID* + auxin) are included for comparison (dataset from Candelli et al., 2018). *nrd1-AID* strain behaves as a hypomorphic allele.

(C) Examples of the meiotic progeny of the indicated diploids strains are shown.

(D) Serial dilution spotting of the indicated strains is shown. *rnh1Δ rnh201Δ* is abbreviated as *rnhΔΔ*.

(E) Serial dilution spotting of the indicated strains is shown. Cells (+*RNH1*) carry *GAL-RNH1* inserted ectopically.

(legend continued on next page)

growth kinetics and sensitivity to both hydroxyurea (HU) and methyl methanesulfonate (MMS). One possibility might be that Sen1 at RFs is redundant with the enzymatic activity of other factors, such as the RNase H1 and H2 enzymes. We crossed *rnh1Δ rnh201Δ* cells with *SEN1 or sen1-3* strains and analyzed their meiotic progeny. Although single deletion of either *RNH1* or *RNH201* combined with *sen1-3* did not present any synthetic defects, *sen1-3 rnh1Δ rnh201Δ* cells were inviable (Figure 4C), similarly to *rnh1Δ rnh201Δ sen1-1* mutants (Figure S3G). Overexpression of *sen1-3* under the strong *ACT1* promoter suppresses the synthetic lethality of *sen1-3* with *rnh1Δ rnh201Δ*, suggesting that higher levels of Sen1 activity can compensate for lack of the specific replisome-tethering mechanism. Yet these cells display growth defects at 37°C, with cells accumulating in G2/M and triggering checkpoint activation (Figures S3H–S3J). Moreover, *ACT1-sen1-3* is unable to suppress the hyper-sensitivity of *rnh1Δ rnh201Δ* to HU and is synthetic defective for MMS sensitivity (Figure 4D). Altogether, these findings suggest that Sen1 at RF might either be redundant with RNases H1 and H2 or become essential to deal with the DNA:RNA hybrids accumulating in the absence of RNase H.

To explore whether increased levels of DNA:RNA hybrids lead to synthetic defects in *sen1-3* cells, we generated *hpr1Δ sen1-3* cells. Hpr1 is a component of the THO complex involved in the processing and export of mRNA (Chávez et al., 2000). *hpr1Δ* mutants accumulate R-loops and show defects in transcription elongation (García-Benítez et al., 2017; Chávez and Aguilera, 1997; Chávez et al., 2000). *hpr1Δ sen1-3* double mutants showed growth defects at higher temperatures and increased sensitivity to replication stress (Figure 4E). To explore whether defects arise during DNA replication, we analyzed the kinetics of Rad52 foci formation in cells released in S phase. The experiment was conducted at permissive temperatures (28°C) as *hpr1Δ* cells failed to synchronously bud at 35°C and 37°C. We observed that *sen1-3* causes a small but statistically significant increase in recombination in late S phase, although *hpr1Δ sen1-3* cells showed synthetic defects and an increase in recombination (Figures 4F and S4A–S4D). Interestingly, the increased rates of recombination and growth defects in *hpr1Δ sen1-3* cells were suppressed by overexpression of *RNH1* (Figures 4E and 4F), thus suggesting that DNA:RNA hybrids are toxic in these mutants.

To directly test the levels of DNA:RNA hybrids, we visualized them in chromosome spreads (Wahba et al., 2011). As previously observed, both *rnh1Δ rnh201Δ* and *hpr1Δ* mutants showed high levels of DNA:RNA hybrids (Figures 4G and S4E; Chan et al., 2014). Surprisingly, we did not observe any increase in the levels of DNA:RNA hybrids in *hpr1Δ sen1-3* cells. Similar results were observed by slot-blot analysis (Figure S4F). Given that phenotypic suppression by RNase H overexpression is accepted as a marker for R-loops, these results suggest that the suppression of *hpr1Δ sen1-3* by overexpression of *RNH1* might occur by removing short or labile DNA:RNA hybrids, not readily detectable by the S9.6 antibody used in our analysis.

sen1-3 Cells Are Defective in Replication Fork Progression and Genome Stability in the Absence of *MRC1*

Because both *sen1-1* and *sen1-3* are synthetically lethal in the absence of *RNH1* and *RNH201*, we wanted to explore whether other pathways, essential for maintaining cell viability in *sen1-1* (Alzu et al., 2012; Mischo et al., 2011), are also important in *sen1-3*. Only a subset of deletion mutants described to negatively affect viability in *sen1-1* cells showed robust defects in cell viability in *sen1-3* cells (summarized in Figure 5A). Namely, we observed temperature sensitivity and increased sensitivity to DNA-damaging agents when *sen1-3* was crossed with either *mrc1Δ*, *ctf18Δ*, or *rad53Δ sml1Δ* (Figure 5B).

Mrc1, Ctf18, and Rad53 are key components of the S phase checkpoint, and all three mutants confer temperature sensitivity in *sen1-3* cells. To further explore the defects of *mrc1Δ sen1-3* mutants, we analyzed the DNA replication dynamics of cells arrested in G1 and then released in S phase at 37°C. The *mrc1Δ sen1-3* cells show a delay during DNA replication and accumulation of cells arrested in G2/M (Figure 5C). Correspondingly, we observed an increase in Rad52-GFP foci accumulating during the later stages of DNA replication, both in the *mrc1Δ sen1-3* and *ctf18Δ sen1-3* cells released in S phase at 37°C (Figures 5D and 5E). In addition, *mrc1Δ sen1-3* and *ctf18Δ sen1-3* cells showed an increase in cells carrying multiple foci of Rad52 (Figure S5A). Similar to what is seen in Figure 4F, we also observed a small but statistically significant increase in Rad52 foci in *sen1-3* mutants compared to wild-type. To determine whether DNA:RNA hybrids contribute to the phenotypes observed in *sen1-3 mrc1Δ*, we repeated the experiments following the overexpression of *RNH1.* This failed to suppress the growth defects and the increase in recombination during S phase (Figures 5F, 5G, and S5B). Similar results were obtained when overexpressing the human ortholog of *RNH1* (Figures S5C and S5D; Wahba et al., 2011; Bonnet et al., 2017).

To analyze whether the increased recombination observed during replication in *sen1-3*, *mrc1Δ sen1-3*, and *hpr1Δ sen1-3* compared to *SEN1* leads to an increase in genomic instability, we measured the rate of direct-repeat recombination using plasmids carrying partially overlapping fragments of the *LEU2* gene separated by 39 or 3,900 nt (plasmids pL and pLYΔNS, respectively) (Mischo et al., 2011; González-Aguilera et al., 2008). We observed, as previously described, that recombination increased with the length of the transcript. Moreover, although *mrc1Δ sen1-3* showed a modest increase in recombination compared to *mrc1Δ* for both plasmids, *hpr1Δ sen1-3* showed greater increases in the rate of recombination (Figure 5H). Furthermore, we tested for defects in mini-chromosome maintenance by transforming a single-copy plasmid carrying an *ADE2* gene and scoring for the rate of plasmid loss in the absence of selective pressure by measuring the rate of white colonies (carrying the plasmid) and red (without the plasmid). Cells carrying the *sen1-3* allele showed higher levels of plasmid loss,

(F) The indicated strains, carrying a *RAD52-GFP* allele with or without the *GAL-RNH1* construct, were grown as shown in Figures S4A–S4D. Samples were taken at the indicated time points, fixed, and analyzed for the presence of Rad52 foci (triplicate biological repeats). n.s., not significant; **$p < 0.05$; ***$p < 0.01$.
(G) The indicated strains were grown to exponential phase at 28°C; DNA:RNA hybrids were analyzed by immunofluorescence of chromosome spreads (triplicate biological repeats). Samples were treated in parallel with RNase H.

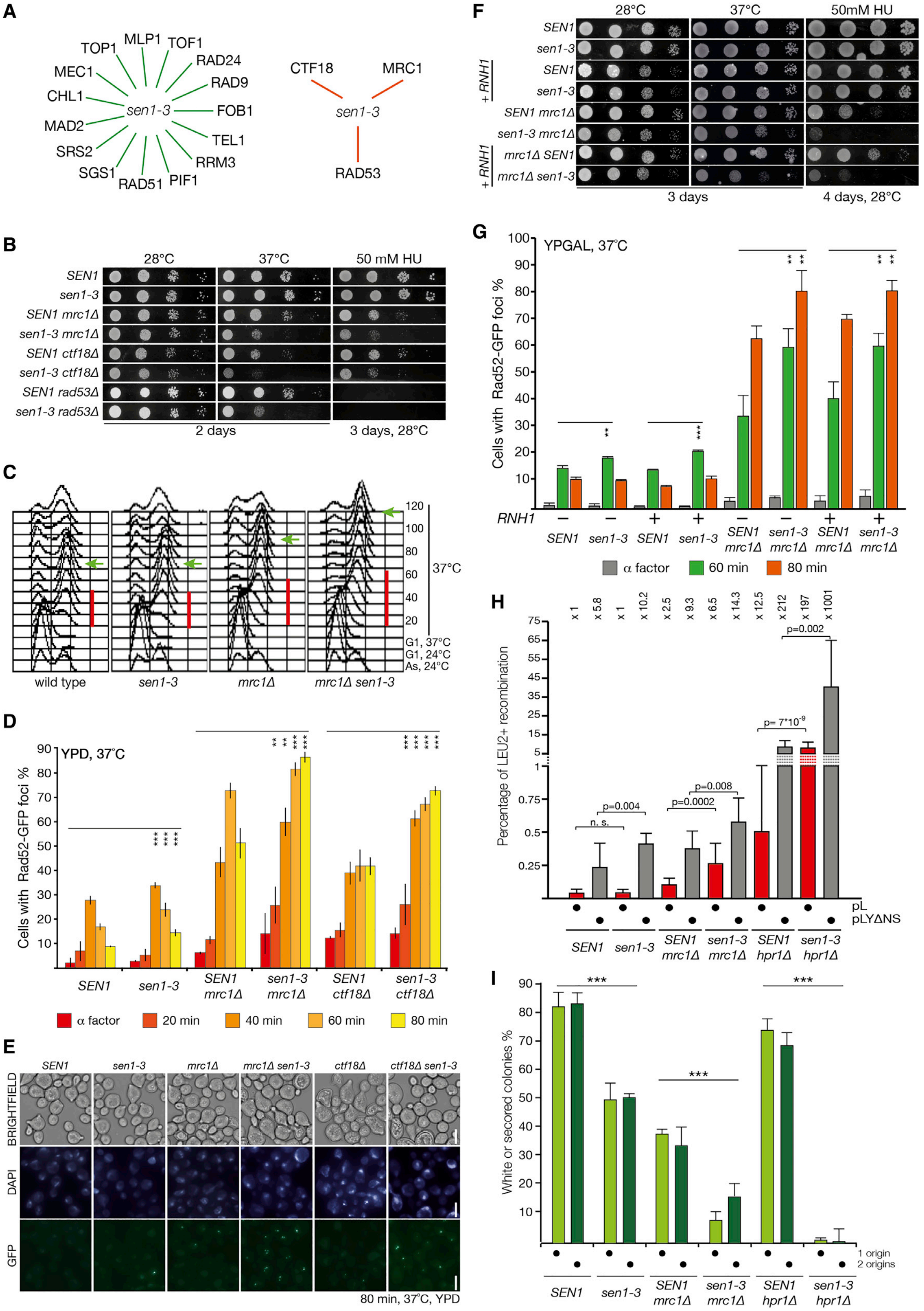

(legend on next page)

exacerbated in the absence of *MRC1* and *HPR1* (Figures 5I and S5E). Strikingly, *sen1-3 hpr1Δ* completely failed to retain the plasmid. The addition of a second origin of replication did not rescue the chromosome maintenance defects, and overexpression of *RNH1* only partially suppressed the defects in *hpr1Δ sen1-3* cells (Figure S5F).

DISCUSSION

Here, we have shown that Sen1 is a bona fide partner of the yeast replisome. The N-terminal domain mediates binding to replisomes, mainly via Ctf4 and Mrc1. Additional binding partners of Sen1 are likely because Sen1 shows some residual interaction with replisomes in the absence of Ctf4 and Mrc1. It is not yet clear whether multiple Sen1 molecules are recruited to RFs by independently binding separate subunits of the replisome with different affinities or whether multiple replisome components coordinately bind a single Sen1 to increase its strength of interaction. IPs of the N-terminal domain of Sen1 suggest a competition between Mrc1 and Ctf4 for Sen1 as Mrc1 binding increases in *ctf4Δ* cells (Figures 2D and 2E). This supports the multiple independent binding hypothesis. However, deletion of either *CTF4* or *MRC1* decreases overall binding of Sen1 to the replisome (Figures 2F, 2H, S2C, and S2D), compatible with a cooperative recruitment of Sen1. Interestingly, the mutation of three amino acids in *sen1-3* abolishes binding to both Ctf4 and Mrc1. Thus, the mutated residues either correspond to the direct interaction site for both Ctf4 and Mrc1 or they cause a change in conformation of a larger section of Sen1, thus affecting two distinct binding surfaces for Ctf4 and Mrc1. Both hypotheses are compelling, and further work is needed to determine which is correct.

The *sen1-3* allele is a separation of function mutant that breaks the interaction with the replisome without affecting the binding to RNAPII or transcription termination (Figures 3E, 4A, and 4B). However, we cannot exclude that *sen1-3* might affect other Sen1 interactions beyond the replisome. In addition, minimal levels of interaction with replisomes might be retained in *sen1-3* cells, thus weakening the severity of the phenotype observed. Nevertheless, this allele provides us with a tool to dissect the function of the helicase at RFs without affecting its catalytic activity and the bulk of its transcription functions.

It has been previously proposed (using the *sen1-1* allele or Sen1 depletion) that Sen1's presence at RFs is required to quickly remove the R-loops accumulating and interfering with RF progression (Alzu et al., 2012; Brambati et al., 2018; Mischo et al., 2011). In our experimental setting, however, loss of Sen1 from RFs did not show increases in DNA:RNA hybrids or dramatic defects in RF progression (Figures 4G and 5C). In fact, the loss of Sen1 from the replisome only leads to modest defects (small increases in post-replicative recombination and instability of mini-chromosomes; Figures 4F, 5D, and 5I). This suggests that when Sen1 is proficient in transcription termination, there might be enough redundancy at the RFs to deal with DNA:RNA hybrids. However, we observe lethality or severe growth defects when the *sen1-3* allele is present in genetic backgrounds with high endogenous levels of R-loops, such as *rnh1Δ rnh201Δ* and *hpr1Δ*. This supports the idea of an important role for Sen1 in dealing with DNA:RNA hybrids at RFs. Surprisingly, we do not observe an increase in DNA:RNA hybrids levels in *sen1-3* and in *hpr1Δ sen1-3* cells (Figures 4G, S4E, and S4F). Moreover, although increased levels of R-loops have been described for *top1Δ* (El Hage et al., 2010), *pif1Δ* (Boulé and Zakian, 2007; Tran et al., 2017), *sgs1Δ* (Chang et al., 2017), or *mlp1Δ* (García-Benítez et al., 2017), these deletions do not show defects in cell viability or DNA damage sensitivity in combination with *sen1-3* (Figure 5A). This suggests that not all increases in R-loops might be necessarily toxic in *sen1-3* cells. One possibility is that different mutations might lead to dissimilar levels or distinct biochemical features of the R-loops. Moreover, different genetic backgrounds might lead to the accumulation of DNA:RNA hybrids at different sites of the genome (as recently observed; Costantino and Koshland, 2018). Therefore, Sen1 association with the replisome might become critical for the timely resolution of some DNA:RNA hybrids in specific circumstances.

The recruitment of Sen1 at RFs also appears to promote DNA replication independently of R-loops. In fact, in *sen1-3* cells, overexpression of *RNH1* fails to suppress the higher levels of recombination observed in *sen1-3* (Figures 4F and 5D). Given the prominent role of Sen1 in transcription termination described in the literature, it is tempting to speculate that Sen1 might remove transcribing or stalled RNA polymerases at RFs (Han et al., 2017; Porrua and Libri, 2013). Alternatively, Sen1 might

Figure 5. *sen1-3* Presents Synthetic Defects with *mrc1Δ*, *ctf18Δ*, and *rad53Δ*, Leading to Increased Recombination and Mini-chromosome Loss

(A) Summary of the genetic interactions tested with the *sen1-3* allele. Some double mutants (orange line) showed marked differences in temperature sensitivity and DNA damage sensitivity although others did not (green line).

(B) Examples of the defects observed with *sen1-3.* Serial dilution spotting of the indicated strains is shown. The double mutant *rad53Δ sml1Δ* is indicated as *rad53Δ*.

(C) The indicated strains were arrested in G1, shifted to 37°C for 1 h, and released in S phase at 37°C. FACS samples were taken at the indicated times. Red bar, length of DNA replication; green arrow, beginning of the end of mitosis.

(D) Cells, carrying a *RAD52-GFP* allele, were treated as in (C). Samples were taken at the indicated time points, fixed, and analyzed for the presence of Rad52 foci (triplicate biological repeats). $^{**}p < 0.05$; $^{***}p < 0.01$.

(E) Examples of the microscopy data of the experiment in (D). Scale bars represent 5 μm.

(F) Serial dilution spotting of the indicated strains is shown. Cells (+*RNH1*) carry an ectopic *GAL1-RNH1* construct.

(G) *RNH1* overexpression does not suppress the increase in recombination in *mrc1Δ sen1-3* cells. Cell cultures were treated as in (C), except they were grown in YPGAL medium (triplicate biological repeats). $^{**}p < 0.05$; $^{***}p < 0.01$.

(H) The *sen1-3* allele causes an increase in recombination. The cells were transformed with the plasmids pL or pLYΔNS. The ratio of the number of the colonies carrying a recombinant plasmid (*LEU2*) over the total number of cells carrying a plasmid (*URA3*) is shown.

(I) Cells were transformed with plasmids carrying an *ADE2* marker and 1 or 2 origins. Percentage of white colonies over the total number of colonies scored is shown (a measure of genome stability; $^{***}p < 0.5\ 10^{-7}$).

be required to remove other barriers to fork progression, or *RNH1* overexpression might not be sufficient to remove DNA:RNA hybrids present at RF with kinetics similar to Sen1, thus leading to increased fork stalling. In either case, we observe that cells rely on the functions of Mrc1 to promote fork progression and minimize DNA recombination in a *sen1-3* background (Figures 5B–5G). Interestingly, we observe that three key mediators and effectors of the S phase checkpoint (*MRC1*, *CTF18*, and *RAD53*) genetically interact with *sen1-3*. We did not observe any synthetic defects between *sen1-3* and either *mec1Δ sml1Δ*, *tel1Δ*, or *mec1Δ sml1Δ tel1Δ* (not shown). This raises the possibility that Mrc1, Ctf18, and Rad53 might be involved in the response to defects arising in *sen1-3* cells independently of Mec1 and Tel1. Alternatively, the synthetic defects observed are the consequence of other deficiencies in these cells, independent of the S phase checkpoint response. For example, Mrc1 has a key role in RF progression (Yeeles et al., 2017; Hodgson et al., 2007; Duch et al., 2018).

Given that eukaryotic orthologs of Sen1 contain an extended non-catalytic N-terminal sequence (the function of which is still largely unknown), it will be interesting to investigate further whether Senataxin or any of its paralogs (Aquarius, IGHMBP2, RENT1, and ZNFx1) associate with replisomes in higher eukaryotes.

STAR★METHODS

Detailed methods are provided in the online version of this paper and include the following:

- KEY RESOURCES TABLE
- LEAD CONTACT AND MATERIALS AVAILABILITY
- EXPERIMENTAL MODEL AND SUBJECT DETAILS
- METHOD DETAILS
  - Yeast Strains and Growth Conditions
  - Cell cycle experiments
  - Harvesting cells for IP
  - Western Blots
  - IP
  - MS Analysis of IPs
  - Counting of Rad52-foci to assess DNA damage
  - Chromosome spreads and microscopy
  - Quantification of R-loops
  - Reverse transcription followed by quantitative PCR
  - Cross-linking and analysis of cDNA (CRAC)
- QUANTIFICATION AND STATISTICAL ANALYSIS
- DATA AND CODE AVAILABILITY

SUPPLEMENTAL INFORMATION

Supplemental Information can be found online at https://doi.org/10.1016/j.celrep.2020.01.087.

ACKNOWLEDGMENTS

We thank Karim Labib, Benoit Palancade, Andres Aguilera, and Nicholas Proudfoot for strains, antibodies, and plasmids. We are grateful to Karim Labib, Jordi Torres-Rosell, Jonathan Millar, and Andrew McAinsh for feedback. The authors acknowledge CAMDU (Computing and Advanced Microscopy Unit) and Media Preparation Facility at the University of Warwick for their assistance in this work, as well as the help of the high-throughput sequencing core facility of I2BC (Centre de Recherche de Gif-sur-Yvette, France). G.D.P. and R.A. were funded by Cancer Research UK Career Development Fellowship C44595/A16326. R.A. has been funded by Chancellor International Scholarship at the University of Warwick. E.C.L. is funded by the BBSRC MIBTP program. This work was also supported by the Centre National de la Recherche Scientifique (CNRS), l'Agence National pour la Recherche (ANR) grants ANR-12-BSV8-0014-01 and ANR-16-CE12-0022-01 to D.L. and the Labex Who Am I? (ANR-11-LABX-0071 et Idex ANR-11-IDEX-0005-02 to D.L. U.A. is supported by a fellowship from the French Ministry of Research.

AUTHOR CONTRIBUTIONS

R.A., D.L., and G.D.P. conceived the study; R.A., E.C.L., U.A., and G.D.P. performed experiments; all authors analyzed experiments; and R.A. and G.D.P. wrote the manuscript. All authors discussed the data and commented and helped improve the manuscript.

DECLARATION OF INTERESTS

The authors declare no competing interests.

Received: March 5, 2019
Revised: September 6, 2019
Accepted: January 24, 2020
Published: February 18, 2020

REFERENCES

Aguilera, A., and García-Muse, T. (2012). R loops: from transcription byproducts to threats to genome stability. Mol. Cell *46*, 115–124.

Alzu, A., Bermejo, R., Begnis, M., Lucca, C., Piccini, D., Carotenuto, W., Saponaro, M., Brambati, A., Cocito, A., Foiani, M., and Liberi, G. (2012). Senataxin associates with replication forks to protect fork integrity across RNA-polymerase-II-transcribed genes. Cell *151*, 835–846.

Arigo, J.T., Eyler, D.E., Carroll, K.L., and Corden, J.L. (2006). Termination of cryptic unstable transcripts is directed by yeast RNA-binding proteins Nrd1 and Nab3. Mol. Cell *23*, 841–851.

Bell, S.P., and Labib, K. (2016). Chromosome duplication in Saccharomyces cerevisiae. Genetics *203*, 1027–1067.

Bonnet, A., Grosso, A.R., Elkaoutari, A., Coleno, E., Presle, A., Sridhara, S.C., Janbon, G., Géli, V., de Almeida, S.F., and Palancade, B. (2017). Introns protect eukaryotic genomes from transcription-associated genetic instability. Mol. Cell *67*, 608–621.e6.

Boulé, J.B., and Zakian, V.A. (2007). The yeast Pif1p DNA helicase preferentially unwinds RNA DNA substrates. Nucleic Acids Res. *35*, 5809–5818.

Brambati, A., Zardoni, L., Achar, Y.J., Piccini, D., Galanti, L., Colosio, A., Foiani, M., and Liberi, G. (2018). Dormant origins and fork protection mechanisms rescue sister forks arrested by transcription. Nucleic Acids Res. *46*, 1227–1239.

Burgers, P.M.J., and Kunkel, T.A. (2017). Eukaryotic DNA replication fork. Annu. Rev. Biochem. *86*, 417–438.

Candelli, T., Challal, D., Briand, J.B., Boulay, J., Porrua, O., Colin, J., and Libri, D. (2018). High-resolution transcription maps reveal the widespread impact of roadblock termination in yeast. EMBO J. *37*, e97490.

Cerritelli, S.M., and Crouch, R.J. (2009). Ribonuclease H: the enzymes in eukaryotes. FEBS J. *276*, 1494–1505.

Chan, Y.A., Aristizabal, M.J., Lu, P.Y., Luo, Z., Hamza, A., Kobor, M.S., Stirling, P.C., and Hieter, P. (2014). Genome-wide profiling of yeast DNA:RNA hybrid prone sites with DRIP-chip. PLoS Genet. *10*, e1004288.

Chang, E.Y., Novoa, C.A., Aristizabal, M.J., Coulombe, Y., Segovia, R., Chaturvedi, R., Shen, Y., Keong, C., Tam, A.S., Jones, S.J.M., et al. (2017).

RECQ-like helicases Sgs1 and BLM regulate R-loop-associated genome instability. J. Cell Biol. *216*, 3991–4005.

Chávez, S., and Aguilera, A. (1997). The yeast *HPR1* gene has a functional role in transcriptional elongation that uncovers a novel source of genome instability. Genes Dev. *11*, 3459–3470.

Chávez, S., Beilharz, T., Rondón, A.G., Erdjument-Bromage, H., Tempst, P., Svejstrup, J.Q., Lithgow, T., and Aguilera, A. (2000). A protein complex containing Tho2, Hpr1, Mft1 and a novel protein, Thp2, connects transcription elongation with mitotic recombination in *Saccharomyces cerevisiae*. EMBO J. *19*, 5824–5834.

Chinchilla, K., Rodriguez-Molina, J.B., Ursic, D., Finkel, J.S., Ansari, A.Z., and Culbertson, M.R. (2012). Interactions of Sen1, Nrd1, and Nab3 with multiple phosphorylated forms of the Rpb1 C-terminal domain in *Saccharomyces cerevisiae*. Eukaryot. Cell *11*, 417–429.

Costantino, L., and Koshland, D. (2018). Genome-wide map of R-loop-induced damage reveals how a subset of R-loops contributes to genomic instability. Mol. Cell *71*, 487–497.e3.

Creamer, T.J., Darby, M.M., Jamonnak, N., Schaughency, P., Hao, H., Wheelan, S.J., and Corden, J.L. (2011). Transcriptome-wide binding sites for components of the Saccharomyces cerevisiae non-poly(A) termination pathway: Nrd1, Nab3, and Sen1. PLoS Genet. *7*, e1002329.

Culbertson, M.R., and Leeds, P.F. (2003). Looking at mRNA decay pathways through the window of molecular evolution. Curr. Opin. Genet. Dev. *13*, 207–214.

De Piccoli, G., Katou, Y., Itoh, T., Nakato, R., Shirahige, K., and Labib, K. (2012). Replisome stability at defective DNA replication forks is independent of S phase checkpoint kinases. Mol. Cell *45*, 696–704.

Duch, A., Canal, B., Barroso, S.I., García-Rubio, M., Seisenbacher, G., Aguilera, A., de Nadal, E., and Posas, F. (2018). Multiple signaling kinases target Mrc1 to prevent genomic instability triggered by transcription-replication conflicts. Nat. Commun. *9*, 379.

El Hage, A., French, S.L., Beyer, A.L., and Tollervey, D. (2010). Loss of Topoisomerase I leads to R-loop-mediated transcriptional blocks during ribosomal RNA synthesis. Genes Dev. *24*, 1546–1558.

El Hage, A., Webb, S., Kerr, A., and Tollervey, D. (2014). Genome-wide distribution of RNA-DNA hybrids identifies RNase H targets in tRNA genes, retrotransposons and mitochondria. PLoS Genet. *10*, e1004716.

Gambus, A., van Deursen, F., Polychronopoulos, D., Foltman, M., Jones, R.C., Edmondson, R.D., Calzada, A., and Labib, K. (2009). A key role for Ctf4 in coupling the MCM2-7 helicase to DNA polymerase α within the eukaryotic replisome. EMBO J. *28*, 2992–3004.

García-Benítez, F., Gaillard, H., and Aguilera, A. (2017). Physical proximity of chromatin to nuclear pores prevents harmful R loop accumulation contributing to maintain genome stability. Proc. Natl. Acad. Sci. USA *114*, 10942–10947.

García-Pichardo, D., Cañas, J.C., García-Rubio, M.L., Gómez-González, B., Rondón, A.G., and Aguilera, A. (2017). Histone mutants separate R loop formation from genome instability induction. Mol. Cell *66*, 597–609.e5.

González-Aguilera, C., Tous, C., Gómez-González, B., Huertas, P., Luna, R., and Aguilera, A. (2008). The THP1-SAC3-SUS1-CDC31 complex works in transcription elongation-mRNA export preventing RNA-mediated genome instability. Mol. Biol. Cell *19*, 4310–4318.

Granneman, S., Kudla, G., Petfalski, E., and Tollervey, D. (2009). Identification of protein binding sites on U3 snoRNA and pre-rRNA by UV cross-linking and high-throughput analysis of cDNAs. Proc. Natl. Acad. Sci. USA *106*, 9613–9618.

Grubb, J., Brown, M.S., and Bishop, D.K. (2015). Surface spreading and immunostaining of yeast chromosomes. J. Vis. Exp., e53081.

Gudipati, R.K., Villa, T., Boulay, J., and Libri, D. (2008). Phosphorylation of the RNA polymerase II C-terminal domain dictates transcription termination choice. Nat. Struct. Mol. Biol. *15*, 786–794.

Hamperl, S., Bocek, M.J., Saldivar, J.C., Swigut, T., and Cimprich, K.A. (2017). Transcription-replication conflict orientation modulates R-loop levels and activates distinct DNA damage responses. Cell *170*, 774–786.e19.

Han, Z., Libri, D., and Porrua, O. (2017). Biochemical characterization of the helicase Sen1 provides new insights into the mechanisms of non-coding transcription termination. Nucleic Acids Res. *45*, 1355–1370.

Hazelbaker, D.Z., Marquardt, S., Wlotzka, W., and Buratowski, S. (2013). Kinetic competition between RNA polymerase II and Sen1-dependent transcription termination. Mol. Cell *49*, 55–66.

Helmrich, A., Ballarino, M., and Tora, L. (2011). Collisions between replication and transcription complexes cause common fragile site instability at the longest human genes. Mol. Cell *44*, 966–977.

Helmrich, A., Ballarino, M., Nudler, E., and Tora, L. (2013). Transcription-replication encounters, consequences and genomic instability. Nat. Struct. Mol. Biol. *20*, 412–418.

Hodgson, B., Calzada, A., and Labib, K. (2007). Mrc1 and Tof1 regulate DNA replication forks in different ways during normal S phase. Mol. Biol. Cell *18*, 3894–3902.

Huertas, P., and Aguilera, A. (2003). Cotranscriptionally formed DNA:RNA hybrids mediate transcription elongation impairment and transcription-associated recombination. Mol. Cell *12*, 711–721.

Janke, C., Magiera, M.M., Rathfelder, N., Taxis, C., Reber, S., Maekawa, H., Moreno-Borchart, A., Doenges, G., Schwob, E., Schiebel, E., and Knop, M. (2004). A versatile toolbox for PCR-based tagging of yeast genes: new fluorescent proteins, more markers and promoter substitution cassettes. Yeast *21*, 947–962.

Jankowsky, E. (2011). RNA helicases at work: binding and rearranging. Trends Biochem. Sci. *36*, 19–29.

Kamimura, Y., Tak, Y.S., Sugino, A., and Araki, H. (2001). Sld3, which interacts with Cdc45 (Sld4), functions for chromosomal DNA replication in *Saccharomyces cerevisiae*. EMBO J. *20*, 2097–2107.

Kanemaki, M., and Labib, K. (2006). Distinct roles for Sld3 and GINS during establishment and progression of eukaryotic DNA replication forks. EMBO J. *25*, 1753–1763.

Kim, M., Krogan, N.J., Vasiljeva, L., Rando, O.J., Nedea, E., Greenblatt, J.F., and Buratowski, S. (2004). The yeast Rat1 exonuclease promotes transcription termination by RNA polymerase II. Nature *432*, 517–522.

Kim, T.S., Liu, C.L., Yassour, M., Holik, J., Friedman, N., Buratowski, S., and Rando, O.J. (2010). RNA polymerase mapping during stress responses reveals widespread nonproductive transcription in yeast. Genome Biol. *11*, R75.

Lang, K.S., Hall, A.N., Merrikh, C.N., Ragheb, M., Tabakh, H., Pollock, A.J., Woodward, J.J., Dreifus, J.E., and Merrikh, H. (2017). Replication-transcription conflicts generate R-loops that orchestrate bacterial stress survival and pathogenesis. Cell *170*, 787–799.e18.

Leonaitė, B., Han, Z., Basquin, J., Bonneau, F., Libri, D., Porrua, O., and Conti, E. (2017). Sen1 has unique structural features grafted on the architecture of the Upf1-like helicase family. EMBO J. *36*, 1590–1604.

Liu, B., and Alberts, B.M. (1995). Head-on collision between a DNA replication apparatus and RNA polymerase transcription complex. Science *267*, 1131–1137.

Luke, B., Panza, A., Redon, S., Iglesias, N., Li, Z., and Lingner, J. (2008). The Rat1p 5′ to 3′ exonuclease degrades telomeric repeat-containing RNA and promotes telomere elongation in *Saccharomyces cerevisiae*. Mol. Cell *32*, 465–477.

Martin-Tumasz, S., and Brow, D.A. (2015). Saccharomyces cerevisiae Sen1 helicase domain exhibits 5′- to 3′- helicase activity with a preference for translocation on DNA rather than RNA. J. Biol. Chem. *290*, 22880–22889.

Mischo, H.E., Gómez-González, B., Grzechnik, P., Rondón, A.G., Wei, W., Steinmetz, L., Aguilera, A., and Proudfoot, N.J. (2011). Yeast Sen1 helicase protects the genome from transcription-associated instability. Mol. Cell *41*, 21–32.

Mischo, H.E., Chun, Y., Harlen, K.M., Smalec, B.M., Dhir, S., Churchman, L.S., and Buratowski, S. (2018). Cell-cycle modulation of transcription termination factor Sen1. Mol. Cell *70*, 312–326.e7.

Nishimura, K., Fukagawa, T., Takisawa, H., Kakimoto, T., and Kanemaki, M. (2009). An auxin-based degron system for the rapid depletion of proteins in nonplant cells. Nat. Methods *6*, 917–922.

Pfeiffer, V., Crittin, J., Grolimund, L., and Lingner, J. (2013). The THO complex component Thp2 counteracts telomeric R-loops and telomere shortening. EMBO J. *32*, 2861–2871.

Porrua, O., and Libri, D. (2013). A bacterial-like mechanism for transcription termination by the Sen1p helicase in budding yeast. Nat. Struct. Mol. Biol. *20*, 884–891.

Porrua, O., Hobor, F., Boulay, J., Kubicek, K., D'Aubenton-Carafa, Y., Gudipati, R.K., Stefl, R., and Libri, D. (2012). In vivo SELEX reveals novel sequence and structural determinants of Nrd1-Nab3-Sen1-dependent transcription termination. EMBO J. *31*, 3935–3948.

Prado, F., and Aguilera, A. (2005). Impairment of replication fork progression mediates RNA polII transcription-associated recombination. EMBO J. *24*, 1267–1276.

Rondón, A.G., Mischo, H.E., Kawauchi, J., and Proudfoot, N.J. (2009). Fail-safe transcriptional termination for protein-coding genes in *S. cerevisiae*. Mol. Cell *36*, 88–98.

Schaughency, P., Merran, J., and Corden, J.L. (2014). Genome-wide mapping of yeast RNA polymerase II termination. PLoS Genet. *10*, e1004632.

Skourti-Stathaki, K., Kamieniarz-Gdula, K., and Proudfoot, N.J. (2014). R-loops induce repressive chromatin marks over mammalian gene terminators. Nature *516*, 436–439.

Steinmetz, E.J., Warren, C.L., Kuehner, J.N., Panbehi, B., Ansari, A.Z., and Brow, D.A. (2006). Genome-wide distribution of yeast RNA polymerase II and its control by Sen1 helicase. Mol. Cell *24*, 735–746.

Takayama, Y., Kamimura, Y., Okawa, M., Muramatsu, S., Sugino, A., and Araki, H. (2003). GINS, a novel multiprotein complex required for chromosomal DNA replication in budding yeast. Genes Dev. *17*, 1153–1165.

Thiebaut, M., Kisseleva-Romanova, E., Rougemaille, M., Boulay, J., and Libri, D. (2006). Transcription termination and nuclear degradation of cryptic unstable transcripts: a role for the nrd1-nab3 pathway in genome surveillance. Mol. Cell *23*, 853–864.

Tran, P.L.T., Pohl, T.J., Chen, C.F., Chan, A., Pott, S., and Zakian, V.A. (2017). PIF1 family DNA helicases suppress R-loop mediated genome instability at tRNA genes. Nat. Commun. *8*, 15025.

Ursic, D., Himmel, K.L., Gurley, K.A., Webb, F., and Culbertson, M.R. (1997). The yeast *SEN1* gene is required for the processing of diverse RNA classes. Nucleic Acids Res. *25*, 4778–4785.

Vasiljeva, L., Kim, M., Mutschler, H., Buratowski, S., and Meinhart, A. (2008). The Nrd1-Nab3-Sen1 termination complex interacts with the Ser5-phosphorylated RNA polymerase II C-terminal domain. Nat. Struct. Mol. Biol. *15*, 795–804.

Wahba, L., Amon, J.D., Koshland, D., and Vuica-Ross, M. (2011). RNase H and multiple RNA biogenesis factors cooperate to prevent RNA:DNA hybrids from generating genome instability. Mol. Cell *44*, 978–988.

Westover, K.D., Bushnell, D.A., and Kornberg, R.D. (2004). Structural basis of transcription: nucleotide selection by rotation in the RNA polymerase II active center. Cell *119*, 481–489.

Yeeles, J.T.P., Janska, A., Early, A., and Diffley, J.F.X. (2017). How the eukaryotic replisome achieves rapid and efficient DNA replication. Mol. Cell *65*, 105–116.

Yüce, Ö., and West, S.C. (2013). Senataxin, defective in the neurodegenerative disorder ataxia with oculomotor apraxia 2, lies at the interface of transcription and the DNA damage response. Mol. Cell. Biol. *33*, 406–417.

STAR★METHODS

KEY RESOURCES TABLE

REAGENT or RESOURCE	SOURCE	IDENTIFIER
Antibodies		
Anti-mouse-HRP	Cell Signaling Technology	#7076; RRID:AB_330924
Anti-sheep-HRP	Sigma	A3415; RRID:AB_258076
Anti-Cdc45	Labib Lab	N/A
Anti-Csm3	Labib Lab	N/A
Anti-Ctf4	Labib Lab	N/A
Anti-Dpb2	Labib Lab	N/A
Anti-FLAG	Sigma	F3165; RRID:AB_259529
Anti-HA (12CA5)	Sigma	11583816001; RRID:AB_514505
Sheep IgG	Sigma	S1265; RRID:AB_261431
Anti-Mcm3	Labib Lab	N/A
Anti-Mcm4	Labib Lab	N/A
Anti-Mcm5	Labib Lab	N/A
Anti-Mcm6	Labib Lab	N/A
Anti-Mrc1	Labib Lab	N/A
Anti-MYC	Sigma	M4439; RRID:AB_439694
Anti-Nrd1	Libri Lab	N/A
Anti Nab3	Libri Lab	N/A
Anti-Psf1	Labib Lab	N/A
Anti-Pob3	Labib Lab	N/A
Anti-Pol1	Labib Lab	N/A
Anti-Pol2	De Piccoli Lab	N/A
Anti-Rad53	Abcam	ab166859; RRID:AB_2801547
Anti-Rpo21	Novus Biologicals	NB200-598SS; RRID:AB_2252678
Anti-Sld5	K. Labib	N/A
Anti-TAP-HRP	Sigma	P1291; RRID:AB_1079562
Anti DNA:RNA hybrids S9.6	Kerafast	ENH001; RRID:AB_2687463
Cy3-conjucated anti-mouse	Jackson laboratories	#115165003; RRID:AB_2338680
Anti-dsDNA	Abcam	ab27156; RRID:AB_470907
Chemicals, Peptides, and Recombinant Proteins		
α-factor	Pepceuticals	N/A
AcTEV protease	Thermo-Fischer	12575015
Calmodulin	Sigma	A6112
Complete protease inhibitor cocktail	Roche	11 836 153 001
Dithiothreitol	Sigma	D0632
Dynabeads	Invitrogen	14302D
Ethidium bromide	Sigma	E1510
Hydroxyurea	Sigma	H8627
Methyl methanesulfonate	Sigma	129925
Propidium iodide	Sigma	P4864
Protease Inhibitor Cocktail	Sigma	P8215
Sodium fluoride	Thermo-Fischer	S299500
Zymolyase	Zymo research	#E1005
RNase H	Invitrogen	#18021071

(*Continued on next page*)

Continued

REAGENT or RESOURCE	SOURCE	IDENTIFIER
Sodium glycerophosphate	Johnson Matthey	170096
Universal Nuclease	Pierce	88700
Critical Commercial Assays		
Amersham ECL Western Blotting Detection Reagent	GE Healthcare	RPN2108
LightCycler® FastStart DNA Master SYBR Green I	Roche	03003230001
MLV-Reverse Transcriptase	Thermofischer	28025013
QuikChange Lightning Site-Directed Mutagenesis Kit	QIAGEN	#210519
Experimental Models: Organisms/Strains		
S. cerevisiae (from W303) CS1*MATa*	Rothstein's lab	N/A
S. cerevisiae (from W303) CS74*MATa pep4Δ::ADE2*	Lab strain	N/A
S. cerevisiae (from W303) CS1125*MATa TAP-SLD5 (kanMX) SEN1-9MYC (hphNT) pep4Δ::URA3 ADE2*	This study	N/A
S. cerevisiae (from W303) CS1126*MATa SEN1-9MYC (hphNT) pep4Δ::URA3 ADE2*	This study	N/A
S. cerevisiae (from W303) CS1187*MATa TAP-SLD5 (kanMX) SEN1-9MYC (hphNT) pep4Δ::URA3 ADE2 ctf4Δ::kanMX*	This study	N/A
S. cerevisiae (from W303) CS1353*MATa SEN1-TAP (kanMX) pep4Δ::ADE2*	This study	N/A
S. cerevisiae (from W303) CS1416*MATa TAP-MCM3 (kanMX) SEN1-9MYC (hphNT) pep4Δ::ADE2*	This study	N/A
S. cerevisiae (from W303) CS1711*MATa TAP-MCM3 (kanMX) GAL1-3HA-ø (LEU2) pep4Δ::ADE2*	This study	N/A
S. cerevisiae (from W303) CS1714*MATa TAP-MCM3 (kanMX) leu2-3,112::GAL1-3HA-SEN1 (2-931) (LEU2) pep4Δ::ADE2*	This study	N/A
S. cerevisiae (from W303) CS1534*MATa TAP-SLD5 (kanMX) SEN1-9MYC (hphNT) pep4Δ::URA3 ADE2 mrc1Δ::hphNT*	This study	N/A
S. cerevisiae (from W303) CS1852*MATa leu2-3,112::GAL1-TAP-ø (LEU2) pep4Δ::ADE2*	This study	N/A
S. cerevisiae (from W303) CS1933*MATa leu2-3,112::GAL1-TAP-SEN1 (1095-2231) (LEU2) pep4Δ::ADE2*	This study	N/A
S. cerevisiae (from W303) CS1941*MATa leu2-3,112::GAL1-TAP-SEN1 (2-2231) (LEU2) pep4Δ::ADE2*	This study	N/A
S. cerevisiae (from W303) CS1942*MATa leu2-3,112::GAL1-TAP-SEN1 (2-1901) (LEU2) pep4Δ::ADE2*	This study	N/A
S. cerevisiae (from W303) CS1943*MATa leu2-3,112::GAL1-TAP-SEN1 (931-2231) (LEU2) pep4Δ::ADE2*	This study	N/A
S. cerevisiae (from W303) CS1956*MATa leu2-3,112::GAL1-TAP-SEN1 (2-1103) (LEU2) pep4Δ::ADE2*	This study	N/A
S. cerevisiae (from W303) CS1957*MATa leu2-3,112::GAL1-TAP-SEN1 (2-931) (LEU2) pep4Δ::ADE2*	This study	N/A
S. cerevisiae (from W303) CS2030*MATa TAP-MCM3 (kanMX) leu2-3,112::GAL1-3HA-SEN1 (2-622) (LEU2) pep4Δ::ADE2*	This study	N/A
S. cerevisiae (from W303) CS2032*MATa TAP-MCM3 (kanMX) leu2-3,112::GAL1-3HA-SEN1 (410-931) (LEU2) pep4Δ::ADE2*	This study	N/A
S. cerevisiae (from W303) CS2056*MATα td-MYC-sen1-1 (klTRP1) GAL1-UBR1 (HISMX) leu2-3,112::GAL1-TAP-ø (LEU2)*	This study	N/A
S. cerevisiae (from W303) CS2058*MATα td-MYC-sen1-1 (klTRP1) GAL1-UBR1 (HISMX) leu2-3,112::GAL1-TAP-SEN1 (2-931) (LEU2)*	This study	N/A
S. cerevisiae (from W303) CS2061*MATα td-MYC-sen1-1 (klTRP1) GAL1-UBR1 (HISMX) leu2-3,112::GAL1-TAP-SEN1 (2-1901) (LEU2)*	This study	N/A
S. cerevisiae (from W303) CS2062*MATα td-MYC-sen1-1 (klTRP1) GAL1-UBR1 (HISMX) leu2-3,112::GAL1-TAP-SEN1 (1095-2231) (LEU2)*	This study	N/A

(*Continued on next page*)

Continued

REAGENT or RESOURCE	SOURCE	IDENTIFIER
S. cerevisiae (from W303) CS2148*MATa TAP-MCM3 (kanMX) leu2-3,112::GAL1-3HA-SEN1 (501-931) (LEU2) pep4Δ::ADE2*	This study	N/A
S. cerevisiae (from W303) CS2150*MATa TAP-MCM3 (kanMX) leu2-3,112::GAL1-3HA-SEN1 (622-931) (LEU2) pep4Δ::ADE2*	This study	N/A
S. cerevisiae (from W303) CS2184*MATα td-MYC-sen1-1 (klTRP1) GAL1-UBR1 (HISMX) leu2-3,112::GAL1-TAP-SEN1 (2-1103) (LEU2)*	This study	N/A
S. cerevisiae (from W303) CS2188*MATα td-MYC-sen1-1 (klTRP1) GAL1-UBR1 (HISMX) leu2-3,112::GAL1-TAP-SEN1 (2-2231) (LEU2)*	This study	N/A
S. cerevisiae (from W303) CS2451*MATα td-MYC-sen1-1 (klTRP1) GAL1-UBR1 (HISMX) leu2-3,112::GAL1-TAP-SEN1 (931-2231) (LEU2)*	This study	N/A
S. cerevisiae (from W303) CS2458*MATa/MATα SEN1/SEN1 (931-2231) (HISMX)*	This study	N/A
S. cerevisiae (from W303) CS2582*MATa sen1Δ::URA3-CP leu2-3,112::ACT1-3HA-SEN1 (931-2231) (LEU2)*	This study	N/A
S. cerevisiae (from W303) CS2584*MATa sen1Δ::URA3-CP leu2-3,112::ACT1-3HA-SEN1 (2-2231) (LEU2)*	This study	N/A
S. cerevisiae (from W303) CS2603*MATa leu2-3,112::GAL1-TAP-SEN1 (2-931) (LEU2) pep4Δ::ADE2 ctf4Δ::kanMX*	This study	N/A
S. cerevisiae (from W303) CS2607*MATa sen1Δ::URA3-CP leu2-3,112::ACT1-3HA-SEN1 (2-2231) W773A E774A W777A (LEU2)*	This study	N/A
S. cerevisiae (from W303) CS2609*MATa sen1Δ::URA3-CP leu2-3,112::ACT1-3HA-SEN1 (2-2231) D850A E851G V852A L853G L854A (LEU2)*	This study	N/A
S. cerevisiae (from W303) CS2611*MATa sen1Δ::URA3-CP leu2-3,112::ACT1-3HA-SEN1 (2-2231) V858A R859A I862A (LEU2)*	This study	N/A
S. cerevisiae (from W303) CS2615*MATa sen1Δ::URA3-CP leu2-3,112::ACT1-3HA-SEN1 (2-2231) D876G D877G V880G (LEU2)*	This study	N/A
S. cerevisiae (from W303) CS2617*MATa sen1Δ::URA3-CP leu2-3,112::ACT1-3HA-SEN1 (2-2231) V746G D747G P748G I749G (LEU2)*	This study	N/A
S. cerevisiae (from W303) CS2623*MATa sen1Δ::URA3-CP leu2-3,112::ACT1-3HA-SEN1 (2-2231) L656A S657A K658A I659A L660 (LEU2)*	This study	N/A
S. cerevisiae (from W303) CS2636*MATa sen1Δ::URA3-CP leu2-3,112::ACT1-3HA-SEN1 (2-2231) L656A S657A K658A I659A L660A (LEU2) NRD1- 9MYC (HIS3MX) pep4Δ:: ADE2 TAP-MCM3 (kanMX)*	This study	N/A
S. cerevisiae (from W303) CS2638*MATa sen1Δ::URA3-CP leu2-3,112::ACT1-3HA-SEN1 (2- 2231)W773A E774A W777A (LEU2) NRD1-9MYC (HIS3MX) pep4Δ:: ADE2 TAP-MCM3 (kanMX)*	This study	N/A
S. cerevisiae (from W303) CS2640*MATa sen1Δ::URA3-CP leu2-3,112::ACT1-3HA-SEN1 (2- 2231) D850A E851G V852A L853G L854A (LEU2) NRD1- 9MYC (HIS3MX) pep4Δ:: ADE2 TAP-MCM3 (kanMX)*	This study	N/A
S. cerevisiae (from W303) CS2642*MATa sen1Δ::URA3-CP leu2-3,112::ACT1-3HA-SEN1 (2- 2231) V746G D747G P748G I749G (LEU2) NRD1-9MYC (HIS3MX) pep4Δ:: ADE2 TAP-MCM3 (kanMX)*	This study	N/A
S. cerevisiae (from W303) CS2669*MATa sen1Δ::URA3-CP leu2-3,112::ACT1-3HA-SEN1 (2-2231) (LEU2) NRD1-9MYC (HIS3MX) pep4Δ:: ADE2 TAP-MCM3 (kanMX)*	This study	N/A
S. cerevisiae (from W303) CS2670*MATa sen1Δ::URA3-CP leu2-3,112::ACT1-3HA-SEN1 (2- 2231) (LEU2) NRD1-9MYC (HIS3MX) pep4Δ:: ADE2*	This study	N/A
S. cerevisiae (from W303) CS2716*MATa sen1Δ::URA3-CP leu2-3,112::ACT1-3HA-SEN1 (2-2231) D876G D877G V880G (LEU2)*	This study	N/A
S. cerevisiae (from W303) CS2718*MATa sen1Δ::URA3-CP leu2-3,112::ACT1-3HA-SEN1 (2-2231) T782G I783G Y784G (LEU2)*	This study	N/A

(*Continued on next page*)

Continued

REAGENT or RESOURCE	SOURCE	IDENTIFIER
S. cerevisiae (from W303) CS2734*MATa rnh1Δ:: hphNT rnh201Δ::HISMX*	Lab strain	N/A
S. cerevisiae (from W303) CS2735*MATα rnh1Δ:: hphNT rnh201Δ::HISMX*	Lab strain	N/A
S. cerevisiae (from W303) CS2791*MATa td-sld3-7 (kanMX) GAL1-UBR1 (HIS3MX) leu2-3,112::GAL1-TAP-SEN1 (2-931) (LEU2+) pep4Δ:: ADE2*	This study	N/A
S. cerevisiae (from W303) CS2808*MATa SEN1-TAP (kanMX)*	This study	N/A
S. cerevisiae (from W303) CS2810*MATa sen1-3-TAP (kanMX)*	This study	N/A
S. cerevisiae (from W303) CS2853*MATa SEN1-TAP (kanMX) pep4Δ:: ADE2*	This study	N/A
S. cerevisiae (from W303) CS2854*MATa sen1-3-TAP (kanMX) pep4Δ:: ADE2*	This study	N/A
S. cerevisiae (from W303) CS2859*MATa SEN1-TAP (kanMX) pep4Δ:: URA3 mrc1Δ::hphNT*	This study	N/A
C *S. cerevisiae* (from W303) S2861*MATa sen1-3-TAP (kanMX) pep4Δ:: URA3 mrc1Δ::hphNT*	This study	N/A
S. cerevisiae (from W303) CS2903*MATa td-sld3-7 (kanMX) GAL1-UBR1 (HIS3MX) leu2-3,112::GAL1-TAP-SEN1 (2-931) (LEU2) pep4Δ:: ADE2 ctf4Δ:: kanMX*	This study	N/A
S. cerevisiae (from W303) CS2938*MATα SEN1-TAP (kanMX) hpr1Δ::kanMX*	This study	N/A
S. cerevisiae (from W303) CS2941*MATα sen1-3-TAP (kanMX) hpr1Δ::kanMX*	This study	N/A
S. cerevisiae (from W303) CS2945*MATa SEN1-TAP (kanMX) sml1Δ::HISMX rad53Δ::ADE2*	This study	N/A
S. cerevisiae (from W303) CS2947*MATa sen1-3-TAP (kanMX) sml1Δ::HISMX rad53Δ::ADE2*	This study	N/A
S. cerevisiae (from W303) CS2955*MATa SEN1-TAP (kanMX) ctf18Δ::klTRP1*	This study	N/A
S. cerevisiae (from W303) CS2957*MATa sen1-3-TAP (kanMX) ctf18Δ::klTRP1*	This study	N/A
S. cerevisiae (from W303) CS3167*MATa leu2-3,112::GAL1-TAP-SEN1 (2-931) (LEU2) pep4Δ::ADE2 psf1-1 (ts)*	This study	N/A
S. cerevisiae (from W303) CS3186*MATa leu2-3,112::GAL1-TAP-SEN1 (2-931) (LEU2) pep4Δ::ADE2 mrc1Δ::hphNT*	This study	N/A
S. cerevisiae (from W303) CS3321*MATα leu2-3,112::GAL1-RNH1 (2-348) (LEU2) SEN1-TAP (kanMX) mrc1Δ::hphNT*	This study	N/A
S. cerevisiae (from W303) CS3322*MATa leu2-3,112::GAL1-RNH1 (2-348) (LEU2) sen1-3-TAP (kanMX) mrc1Δ::hphNT*	This study	N/A
S. cerevisiae (from W303) CS3499*MATa SEN1-TAP (kanMX) pep4Δ::ADE2 ctf4Δ::kanMX mrc1-3IAA (HISMX) ADH1-OsTIR1 (klTRP1,URA3)*	This study	N/A
S. cerevisiae (from W303) CS3545*MATa sen1Δ::URA3-CP leu2-3,112::ACT1-3HA-sen1-3 (2-2231) (LEU2) rnh1Δ:: hphNT rnh201Δ::HISMX*	This study	N/A
S. cerevisiae (from W303) CS3547*MATa sen1Δ::URA3-CP leu2-3,112::ACT1-3HA-SEN1 (2-2231) (LEU2) rnh1Δ:: hphNT rnh201Δ::HISMX*	This study	N/A
S. cerevisiae (from W303) CS3562*MATa SEN1-TAP (kanMX) pep4Δ::ADE2 ctf4Δ::kanMX*	This study	N/A
S. cerevisiae (from W303) CS3662*MATa SEN1-TAP (kanMX) mrc1Δ::hphNT leu2-3,112::GAL1-RNH1 (2-348) (LEU2+)*	This study	N/A
S. cerevisiae (from W303) CS3664*MATa sen1-3-TAP (kanMX) mrc1Δ::hphNT leu2-3,112::GAL1-RNH1 (2-348) (LEU2)*	This study	N/A

(*Continued on next page*)

Continued

REAGENT or RESOURCE	SOURCE	IDENTIFIER
S. cerevisiae (from W303) CS3702*MATa TAP-SLD5 (kanMX) SEN1-9MYC (hphNT) pep4Δ::URA3 ADE2 ctf4Δ::kanMX mrc1-3IAA (HISMX) ADH1-OsTIR1 (klTRP1,URA3)*	This study	N/A
S. cerevisiae (from W303) CS3731*MATα SEN1-TAP (kanMX) leu2-3,112::GAL1-RNH1 (2-348) (LEU2)*	This study	N/A
S. cerevisiae (from W303) CS3733*MATa sen1-3-TAP (kanMX) leu2-3,112::GAL1-RNH1 (2-348) (LEU2)*	This study	N/A
S. cerevisiae (from W303) CS3796*MATa SEN1-TAP (kanMX) mad2Δ::kanMX*	This study	N/A
S. cerevisiae (from W303) CS3797*MATα sen1-3-TAP (kanMX) mad2Δ::kanMX*	This study	N/A
S. cerevisiae (from W303) CS3903*MATa leu2-3,112::GAL1-RNH1 (2-348) (LEU2) SEN1-TAP (kanMX) hpr1Δ::kanMX*	This study	N/A
S. cerevisiae (from W303) CS3905*MATa leu2-3,112::GAL1-RNH1 (2-348) (LEU2) sen1-3-TAP (kanMX) hpr1Δ::kanMX*	This study	N/A
S. cerevisiae (from W303) CS4296*MATa SEN1-TAP (kanMX) chl1Δ::kanMX*	This study	N/A
S. cerevisiae (from W303) CS4298*MATα sen1-3-TAP (kanMX) chl1Δ::kanMX*	This study	N/A
S. cerevisiae (from W303) CS4312*MATa NRD1-TAP (kanMX) SEN1-9MYC (hphNT) pep4Δ::URA3-CP ADE2*	This study	N/A
S. cerevisiae (from W303) CS4314*MATa SEN1-TAP (kanMX) pep4Δ::ADE2 rpb1-1 (ts)*	This study	N/A
S. cerevisiae (from W303) DLY2057*MATa sen1-1 (ts)*	Lab strain	N/A
S. cerevisiae (from W303) DLY2281*MATa upf1Δ::TAP::klTRP1*	Lab strain	N/A
S. cerevisiae (from W303) DLY3111*MATa sen1-1 (ts) upf1Δ::TAP::klTRP1*	This study	N/A
S. cerevisiae (from W303) DLY3190*MATa SEN1-TAP (kanMX) upf1Δ::TAP::klTRP1*	This study	N/A
S. cerevisiae (from W303) DLY3191*MATa sen1-3-TAP (kanMX) upf1Δ::TAP::klTRP1*	This study	N/A
Oligonucleotides		
DL377ATGTTCCCAGGTATTGCCGA	This study	N/A
DL378ACACTTGTGGTGAACGATAG	This study	N/A
DL474GCAAAGATCTGTATGAAAGG	This study	N/A
DL475CGCAGAGTTCTTACCAAACG	This study	N/A
DL481TAAATGGCCAACCGCTGTTG	This study	N/A
DL482CCAGCGTACTGCACGCCAGG	This study	N/A
DL1119AAGTGACGAAGTTCATGCTA	This study	N/A
DL1120TCCGTGTCTCTTGTCCTGCA	This study	N/A
Recombinant DNA		
pYM-N24	Janke et al., 2004	Euroscarf
pCS14pRS305-*GAL1-TAP-Ø*	This study	N/A
pCS25pRS305-*GAL1-3HA-Ø*	This study	N/A
pCS26pRS305-*GAL1-3HA-SEN1 (2-931)*	This study	N/A
pCS30pRS305-*GAL1-TAP-SEN1 (2-931)*	This study	N/A
pCS31pRS305-*GAL1-TAP-SEN1 (2-1103)*	This study	N/A
pCS32pRS305-*GAL1-TAP-SEN1 (931-2231)*	This study	N/A
pCS33pRS305-*GAL1-TAP-SEN1 (1095-2231)*	This study	N/A
pCS39pRS305-*GAL1-TAP-SEN1 (2-2231)*	This study	N/A
pCS40pRS305-*GAL1-TAP-SEN1 (2-1901)*	This study	N/A
pCS42pRS305-*GAL1-3HA-SEN1 (2-622)*	This study	N/A

(*Continued on next page*)

Continued

REAGENT or RESOURCE	SOURCE	IDENTIFIER
pCS43pRS305-*GAL1-3HA-SEN1 (410-931)*	This study	N/A
pCS59pRS305-*GAL1-3HA-SEN1 (501-931)*	This study	N/A
pCS61pRS305-*GAL1-3HA-SEN1 (622-931)*	This study	N/A
pCS118pRS305-*ACT1-3HA-SEN1 (931-2231)*	This study	N/A
pCS120pRS305-*ACT1-3HA-SEN1 (2-2231)*	This study	N/A
pCS123pRS305-*ACT1-3HA-SEN1 (2-2231) W773A E774A W777A*	This study	N/A
pCS124pRS305-*ACT1-3HA-SEN1 (2-2231) L656A S657A K658A I659A L660A*	This study	N/A
pCS125pRS305-*ACT1-3HA-SEN1 (2-2231) D850A E851G V852A L853G L854A*	This study	N/A
pCS127pRS305-*ACT1-3HA-SEN1 (2-2231) D876G D877G V880G*	This study	N/A
pCS128pRS305-*ACT1-3HA-SEN1 (2-2231) V746G D747G P748G I749G*	This study	N/A
pCS129pRS305-*ACT1-3HA-SEN1 (2-2231) T782G I783G Y784G*	This study	N/A
pCS188pRS305-*GAL1-RNH1 (2-348)*	This study	N/A
pCS196pRS424-*GPD-hsRNASEH1* (2-286)	From Palancade's lab	N/A
pCS197pRS315-*ADE2*	This study	N/A
pCS198pRS315-*ADE2-ARS306*	This study	N/A
pLpRS316-*leu2Δ3′-39bp-leu2Δ5′*	From Aguilera's lab	N/A
pLYΔNSpRS316-*leu2Δ3′-3900bp-leu2Δ5′*	From Aguilera's lab	N/A
Software and Algorithms		
Excel	Microsoft	RRID:SCR_016137
Illustrator	Adobe	RRID:SCR_014198
ImageJ	NIH	https://imagej.nih.gov/ij/; RRID:SCR_003070
Photoshop	Adobe	RRID:SCR_014199
PredictProtein.org		https://www.predictprotein.org/
RStudio	RStudio	RRID:SCR_000432

LEAD CONTACT AND MATERIALS AVAILABILITY

Further information and requests for resources and reagents should be directed and will be fulfilled by the Lead Contact, Dr Giacomo De Piccoli (g.de-piccoli@warwick.ac.uk). All unique/stable reagents generated in this study are available from the Lead Contact with a completed Materials Transfer Agreement.

EXPERIMENTAL MODEL AND SUBJECT DETAILS

Saccharomyces cerevisiae is the experimental model used in this study. All strains are isogenic to W303, and are listed in the Key Resources Table.

METHOD DETAILS

Yeast Strains and Growth Conditions

All yeasts were grown in YP medium supplemented with either glucose (YPD) or galactose (YPGAL) or raffinose (YPRAF) to a final concentration of 2% (w/v). For solid media, the same formulation was used, but with a final concentration of 1% (w/v) agar. Yeasts were grown at 24, 28, 30 and 37°C, depending on their viability at the different temperatures and as required by the experimental design. For all experiments, the control and test strains were subjected to the same conditions, including temperature.

For cell spotting experiments, cells were grown on non-selective media until colonies were judged to be sufficiently big. Five discrete colonies from individual strains were added to sterile deionised water to create a cell suspension. From this suspension, serial dilutions (0.5 x10^6, 0.5 x10^5, 0.5 x10^4 and 0.5 x10^3 cells/ml) were generated. 10 μL of each suspension was pipetted onto the appropriate media and grown for up to 5 days at the required temperatures.

To assess the genetic interaction between two or three genes, parents carrying the appropriate alleles were first crossed. Analysis of the meiotic progeny was conducted by inducing sporulation of the diploid strains in sporulation medium for 3-5 days at 24°C. Asci

were treated with a 1:10 dilution of β-glucoronidase from *Helix pomatia* (Sigma) for 30 minutes, followed by tetrad dissection onto a YPD plate using a Singer MSM400 micromanipulator. Plates were incubated for 3-4 days at the appropriate temperature.

For the plasmid recombination assay, eight independent clones carrying the appropriate plasmid (pL or pLYΔNS) were each plated in medium lacking leucine (to select for recombination) or lacking uracil (marker for the presence of the plasmid) at 24°C. The experiment was repeated in triplicate. For plasmid loss assays, cells were transformed with the required plasmid (pCS197 or pCS198) and plated on minimum medium lacking leucine and incubated at 24°C. Colonies were left to grow until single isolated colonies were sufficiently big. Five to seven colonies for each strain were then picked, resuspended in sterile water and counted. Around 200-150 cells were then plated onto YPD and incubated at 24°C until red/white coloring was clearly visible. Cells were then incubated at 4°C for three days. We considered white and sectored colonies as white while only fully red colonies were scored as red. The experiment was repeated twice. The plasmid loss assay with or without *GAL-RNH1* was conducted in a similar manner, except that cells were grown and transformed in medium containing galactose and selected in medium lacking adenine (*LEU2* is the reporter gene for the *GAL1-RNH1* construct). Colonies were grown for longer periods of time before colonies were sufficient size big and were plated onto non-selective medium containing galactose.

Cell cycle experiments

Cells were diluted from an inoculum to a density of 0.35×10^7 cells/ml in a suitable volume and left to grow to a final density of 0.7×10^7 cells/ml. The cells were then synchronized in G1 by adding α-factor to a final concentration of 7.5 μg/ml. After the first 90 min, α-factor was added every 30 min to a 3.25 μg/ml final concentration to maintain the cells in G1. When the cultures were shifted to 37°C, cells were spun down and resuspended in pre-warmed medium containing 7.5 μg/ml α-factor. Cells were released from the arrest by washing the cells twice with medium without α-factor. In all experiments in which cells were collected for IPs, cells were grown at 24°C and released into S phase for 30 min, unless stated otherwise in the figure legend. For expressing constructs under the *GAL1* promoter, strains were grown in YPRAF, arrested in G1 using α-factor, upon which YPGAL was substituted for YPRAF. Alternatively, YPGAL was used throughout the experiment (appropriate for constructs that were labile).

Harvesting cells for IP

Harvested cells were immediately cooled to 4°C by washing with an ice-cold solution of HEPES-KOH (pH 7.9), followed by a wash in a solution of 100 mM HEPES-KOH (pH 7.9), 50 mM potassium acetate, 10 mM magnesium acetate and 2 mM EDTA-KOH, still at 4°C. After the wash, the solution was discarded and the cells were re-suspended in a fresh quantity of the same solution supplemented with protease and phosphatase inhibitors, so that the ratio of wet mass of the cells to the final mass of the suspension was either 1:4 (for 250 mL cultures) or 4:5 (for 1 l cultures). The re-suspended cells were immediately flash-frozen by pipetting into a flask holding liquid nitrogen. The frozen cells were kept at −80°C until use for IP. Before freezing, some cells were fixed in 70% (v/v) ethanol to test that cells did not progress through the cell cycle during sample preparation.

For cells with inducible constructs, cultures were grown as described above in YPRAF. After the cells were arrested in G1, the culture was substituted with YPGAL (supplemented with α-factor) to induce transcription from the *GAL1* promoter. Harvesting of G1 cultures can be performed prior to or after induction according to the experimental setup. After 35 min or 1 h of induction, the cells were released in S phase as described above and harvested either 30 min (24°C) or 20 min (30°C or 37°C) post-release. For temperature-sensitive strains or strains tagged with a temperature-degron (e.g *psf1-1*, *td-sld3-7* and *rnh1Δ rnh201Δ ACT1-sen1-3*), the strains were grown and synchronized in G1 at 24°C as described above. Once synchronized and, (optionally) constructs transcriptionally induced, the cells were shifted to 37°C for 1 h. α-factor was added every 20 min to maintain the cells in G1 to a final concentration of 7.5 μg/ml for 1 h. Synchronicity was monitored visually using a microscope and by harvesting a 1 mL sample of the culture by fixing in 70% (v/v) ethanol for flow-cytometric analysis. The cells were then washed and released in S phase. The cells were harvested 20 min after release, including for the *psf1-1* strains that do not actually undergo DNA replication at 37°C as the GINS complex is destabilized. For crosslinking IPs, cells cultures were incubated with formaldehyde for 25 min and treated as in De Piccoli et al. (2012).

Western Blots

Protein samples (TCA-precipitated and non-treated cell extracts, as well as IPs) were run on 5, 6, 7, 8 or 10% polyacrylamide gels. The protein bands were then transferred onto nitrocellulose or PVDF membranes. The proteins bands were then probed with the appropriate primary antibodies for 1 h in a solution of 5% (w/v) skimmed milk in TBST, washed thrice for 5 min in fresh TBST, probed with the appropriate HRP-bound secondary antibody (if any, refer to Key Resources Table) and washed thrice again for 5 min in fresh TBST. The membrane was then treated with the western blotting reagents and the resulting chemiluminescent signal was captured using either films or a digital camera (G:BOX, Chemi XRG, Syngene).

IP

IPs were conducted as previously described (De Piccoli et al., 2012). In brief, cells previously harvested were lysed using a mechanised pestle and mortar at −80°C (Spex Sample Prep, 6870). 1 g of lysate is considered equivalent to 1 ml. To 1 volume of thawed lysate, ¼ volume of a solution of 50% (v/v) glycerol, 100 mM HEPES-KOH (pH 7.9), 50 mM potassium acetate, 50 mM magnesium acetate, 0.5% (v/v) Igepal® CA-630, 2mM EDTA supplemented with protease and phosphatase inhibitors was added. Pierce

Universal Nuclease was added to a final concentration of 0.4 U/μl and samples were left on a rotating platform at 4°C for 30 min. After incubation, the sample was clarified by stepwise centrifugation at 18,700*g* and then at 126,600*g*. The supernatant was isolated, 50 μL of which was added to 100 μL of 1.5 x Laemmli buffer (cell extract). The remaining cell extract was incubated with 100 μL of TAP-beads for 2 h (M-270 Dynabeads® Epoxy beads bound to an anti-sheep IgG). Beads were washed with solutions of 100 mM HEPES-KOH (pH 7.9), 50 mM potassium acetate, 50 mM magnesium acetate, 2 mM EDTA, 0.1% (v/v) Igepal® CA-630 thrice. After washing, 100 μL of 1 x Laemmli buffer was added to the 100 μL of TAP-beads and boiled for 4 min. Crosslinking IPs were conducted as in De Piccoli et al. (2012).

When scaling up was necessary (using 1 l of cells instead of 250 ml), a few changes were implemented to the protocol. Notably, the concentration of the Pierce Universal Nuclease was increased four-fold to a final concentration of 1.6 U/μl and incubation with the nuclease was increased from 30 to 40 min.

MS Analysis of IPs

The samples were processed as above. Following the washes, TAP-Sen1 (2-931) protein was released by using the AcTEV® protease at 24°C for 2 h. Thereafter, the resultant CBP-Sen1 (2-931) (CBP: calmodulin-binding protein) and its specific interactors were incubated with pre-washed calmodulin beads at 4°C for 2 h. After washing, 30 μL of 1 X Laemmli was added to the calmodulin beads and boiled for 4 min. The samples from the four biological replicates were pooled together, flash-frozen on dry ice and stored at −80°C. The samples were then run on commercially sourced 4%–12% acrylamide gel for a short distance (~1 cm). The gel was then cut in thin slices and processed and analyzed by MS Bioworks, USA.

Counting of Rad52-foci to assess DNA damage

Cells carrying the *RAD52-GFP* allele were first grown in liquid medium and synchronized in G1. Cells were released and harvested at different times after release, corresponding to different phases of the cell cycle. Paraformaldehyde was added to the cell suspensions to a final concentration of 3% (w/v) and the samples were incubated at room temperature for 10 min. The cells were then washed with PBS at room temperature. Finally, the samples were re-suspended in fresh PBS and kept at 4°C overnight.

Less than 24 h after fixation (to minimize signal lost due to alteration of the GFP protein), the samples were re-suspended in 500 μL of fresh PBS to which the DNA stain DAPI was added to a final concentration of 1 μg/ml. The samples were incubated at room temperature for 10 min to allow for staining of the DNA. The cells were then washed with PBS to improve the signal-to-noise ratio of the DAPI staining. The cells were then brought to a suitable dilution prior to pipetting on a glass slide onto which a coverslip is applied. Images of cells were acquired (brightfield, ~510 nm emission (GFP), ~460 nm (DAPI)) using a Personal DeltaVision (Applied Precision). The images were analyzed using ImageJ and the number of Rad52-foci were counted. An average of three experiments is shown in the figures.

Chromosome spreads and microscopy

Chromosome spreads were performed as previously described (Wahba et al., 2011; Grubb et al., 2015). Exponentially growing asynchronous cultures were grown in YPD at 28°C. $2x10^8$ cells were harvested and spheroplasted (0.1M potassium phosphate (pH 7.4), 1.2 M sorbitol, 0.5 mM MgCl2, 40 mM DTT, 20 U zymolyase at 30°C for 1 h or until > 90% of cells lysed following addition of 2% sarcosyl. Cells were then washed and resuspended in ice cold 1 M sorbitol (pH 6.4), 0.1 M MES, 0.5 mM MgCl2, 1 mM EDTA to stop spheroplasting reaction. 20 μL of cell suspension was placed onto a slide, followed by 40 μL of fixative (4% paraformaldehyde (w/v), 3.4% sucrose (w/v)), then lysed using 80 μL of 1% lipsol (v/v) for 2 min, followed by addition of 80 μL of fixative and spread across the surface of the slide to dry overnight. Slides pre-treated with RNase H were incubated with 4U of RNase H diluted in 400 μL of 5mg/ml BSA for 1 h at 37°C prior to immunostaining. Slides were immunostained for DNA:RNA hybrids using mouse monoclonal antibody S.96 (Kerafast) diluted 1:2000 (0.25 μg/ml) in blocking buffer (5% BSA, 0.2% milk, 1XPBS) for 1 h. The secondary antibody, Cy3-conjucated goat anti-mouse (Jackson laboratories) was diluted 1:2000 in blocking buffer and incubated in the dark for 1 h. Indirect immunofluorescence was observed using a Deltavision 1 microscope with a 100 × /NA 1.4 objective. Image analysis was performed using ImageJ and Adobe Photoshop. An average of three experiments is shown in the figures.

Quantification of R-loops

Cells growing in liquid culture was harvested and re-suspended in lysis solution (100 mM NaCl, 10 mM Tris-HCl pH 8.0, 1 mM EDTA, 3% (w/v) SDS). To a volume of cell suspension, an equal volume of phenol/chloroform/isoamyl alcohol (25:24:1) (Acros Organics) and another volume of nuclease-free deionised water were added. The cells were then lysed mechanically using glass beads and DNA was isolated by incubating the soluble cell extract to ethanol to a final concentration of 70% (v/v). The DNA was washed with fresh ethanol and re-suspended in nuclease-free TE supplemented with 50 μg/ml RNase A and incubated at 37°C for 1 h only.

The concentration of genomic material was estimated by measuring absorbance at 260 nm. For each sample, 1 μg/μl, 0.5 μg/μl and 0.25 μg/μl dilutions of DNA was prepared, using nuclease-free water. 2 μL of each dilution was treated with either 1U of RNaseH (Invitrogen, #18021071) or 1 U of RNaseH and 1 U of RNase III (Invitrogen, #AM2290) with similar results at 37°C for 1h. As a control, untreated samples were also incubated at 37°C for 1 h. The remaining DNA was then added to 200 μL of 2 X SSC hybridization buffer (0.3M NaCl, 30mM trisodium Citrate, pH 7.0) and transferred to a pre-equilibrated hybond-N+ nylon membrane (GE healthcare, #RPN203B) under vacuum. The DNA was cross-linked to the membrane using UV prior to blocking in either 5% (w/v) milk (anti-R

loops) or 5% (w/v) BSA (anti ds-DNA) at 24°C for 1 h. The membranes were then incubated overnight in 5% milk supplemented the primary antibody at 4°C overnight. After thrice washing in TBST for 30 min, the membrane was incubated with anti-mouse IgG-HRP at 24°C for 1 h. The membranes were treated with ECL, and chemiluminescent signal was visualized using a camera (G:BOX, Chemi XRG, Syngene).

Reverse transcription followed by quantitative PCR

Cells were grown to exponential phase and incubated at permissive (24°C) or non-permissive temperatures (37°C) for 3 h to induce the *sen1-1* phenotype before collection. Analysis was performed in parallel in an *upf1Δ* background for detecting elongated RNA species derived from termination failure that might be degraded in the cytoplasm. The ratio of the read-through fraction over the total amount of *SNR13* RNA is shown as a proxy of transcription termination levels. The mean of three experiments is shown. Error bars represent the standard deviation. Cells were grown in logarithmic phase, and 6 OD_{600} worth of cells were pelleted. Total RNAs were extracted by resuspending cell pellets in 1 volume of acidic phenol (pH 4.3) supplemented with 1 volume of AES Buffer (50 mM Sodium Acetate pH 5.5, 10 mM EDTA, 1% SDS). Mixtures were incubated at 70°C with agitation (1,400 rpm) for 30 min in a thermomixer (Eppendorf), before being centrifuged at *20,000 g* at 4°C for 10 min. Aqueous phases were recovered and subjected to one extra round of hot acidic phenol extraction, followed by one round of chloroform extraction. Total RNAs were finally precipitated with absolute ethanol and sodium acetate pH 5.5, washed once with 70% Ethanol, dried on a SpeedVac (Thermo) and resuspended in 30 μL of RNase-free H_2O. 60-120 μg of total RNAs were recovered routinely.

Reverse transcription was performed using random hexamer-primers annealing at multiple *loci* in the *S. cerevisiae* genome and with oligos dT. 4 μg of total RNAs were mixed to 200 ng of random hexamers and 0.5 μM of oligos dT in a 20 μL reaction containing 50 mM Tris-HCl pH 8.3, 75 mM KCl, 3 mM MgCl2 and 5 mM DTT. Samples were first incubated for 15 min at 70°C to allow RNA denaturation. Then temperature was slowly decreased to 37°C to allow annealing of primers. Lastly, synthesis of cDNAs was performed by adding 200 units of MLV-reverse transcriptase for 45 min at 37°C.

To assess the amount of cDNAs reverse transcribed, quantitative PCR (qPCR) was carried out using two different primer pairs for each target (*SNR13, NEL250c, ACT1*). These allowed the amplification of a product covering either ~300 bp of the 3′ end of *ACT1* (DL377/DL378 primer pair) or ~70 bp in the read-through region of *SNR13* (DL1119/DL1120 primer pair) or ~140 bp in the body of *NEL025c* (DL474/DL475 primer pair) or ~70 bp in the read-though region of *NEL025c* (DL481/DL482 primer pair). qPCR was performed in a 10 μL reaction by mixing 2 μL of the reverse transcribed cDNAs to 5 μL of LightCycler® 480 SYBR Green I Master and 2.5 pmol of both the forward and the reverse primer.

Cross-linking and analysis of cDNA (CRAC)

The CRAC protocol used in this study is derived from Granneman et al. (2009) with a few modifications as described in Candelli et al. (2018). Raw data processing has been performed as described in Candelli et al. (2018). Metagene analysis has been performed as follows: for the CUTs presented in Table S1, we retrieved the polymerase reads count at every position around the features (3′ or 5′ end) and plotted the mean over all the values for these positions in the final aggregate plot. Analysis has been performed in the R Studio environment.

QUANTIFICATION AND STATISTICAL ANALYSIS

Where applicable, data was presented as the average ± standard deviation. t tests were used to compare population means. Statistically significant differences were indicated as such by indicating the value range of the p values.

DATA AND CODE AVAILABILITY

The published article includes all datasets generated or analyzed during this study. The raw data of the metagene analysis of the CUTs shown in Figure 4B are included in Table S1. This study did not generate any unique code.

Cell Reports, Volume *30*

Supplemental Information

Sen1 Is Recruited to Replication Forks via Ctf4 and Mrc1 and Promotes Genome Stability

Rowin Appanah, Emma Claire Lones, Umberto Aiello, Domenico Libri, and Giacomo De Piccoli

A)

Protein ID	MW (kDa)	TAGGED (peptide N)	UNTAGGED (peptide N)
CTF4	104	365	0
MCM4	115	123	0
POL2	256	97	0
SEN1	253	96	0
TOP1	90	85	0
DPB3	59	83	17
MCM3	108	61	0
SLD5	40	34	3
MCM6	113	34	0
DPB2	61	27	6
MRC1	124	25	0
MCM5	86	24	0
HHF1	11	22	0
TOF1	141	22	0

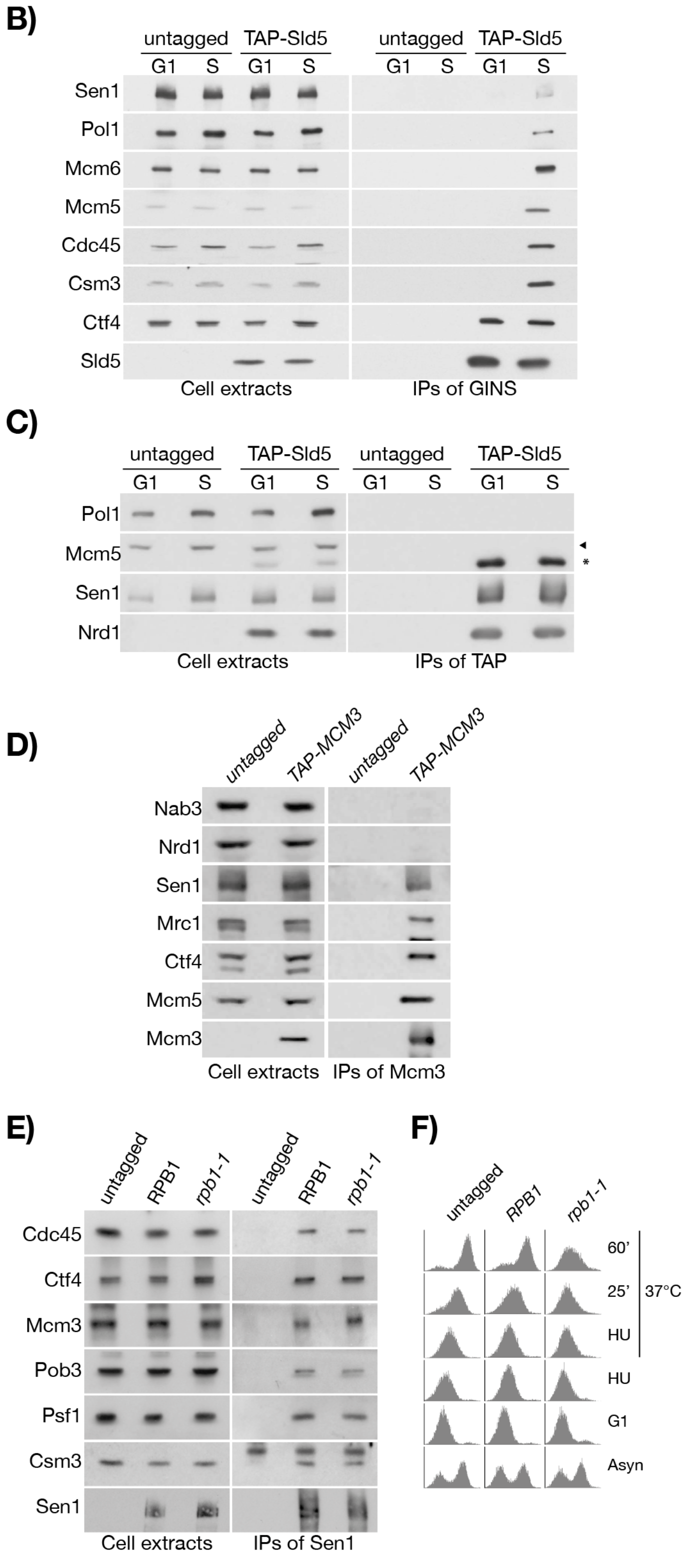

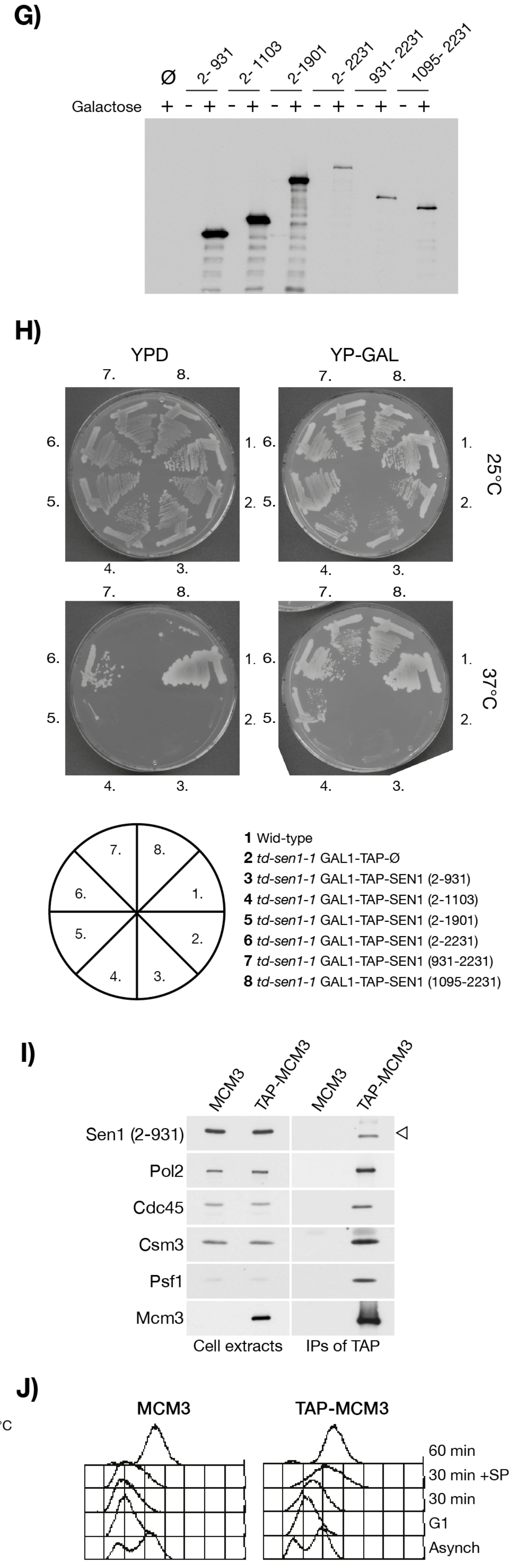

Figure S1. The replisome binds Sen1 in S phase (related to Fig 1)**. A)** Example of the mass spectrometry analysis obtained from the double purification of Sld5 and Mcm4. **B)** Cells carrying the *SEN1-9MYC* allele with a *SLD5* or *TAP-SLD5* allele were synchronously released from G1 into S phase for 30 min at 24°C. Cell extracts were incubated with anti-TAP beads and analysed by immunoblotting. **C)** Nrd1 does not interact with the replisome. *NRD1* or *NRD1-TAP* cells were released from G1 arrest into S phase for 30 min at 24°C. Cell extracts were incubated with anti-TAP beads and analysed by immunoblotting. **D)** Mcm3 immunoprecipitates Sen1 but neither Nrd1 nor Nab3. *MCM3* or *TAP-MCM3* cells were arrested in G1 and released into S phase for 30 min at 24˚C. Cell extracts were incubated with anti-TAP beads and analysed by immunoblotting. **E)** Sen1 interacts *in vivo* with the replisome independently of RNAPII transcription. Wild type or *rpb1-1* cells, either carrying an untagged or TAP-tagged allele of *SEN1*, were arrested in G1 and released in medium containing 0.2 M HU for 75 min at 24˚C. Cultures were then shifted to 37˚C for 1 h. Inactivation of *rpb1-1* cells at 37˚C for 1 h has been shown to lead to a substantial loss of Rpb1-1 from chromatin (Zanton and Pugh, 2006; Kim *et al.*, 2010), to a loss of elongation factors Spt5 and Spt16 (Tardiff, Abruzzi and Rosbash, 2007), to a loss of Sen1 recruitment at highly transcribed genes (Alzu *et al.*, 2012) and to the termination of transcription (Nonet *et al.*, 1987). Cells were then released for 25 min at 37˚C in fresh medium so to allow the synthesis of the bulk of the DNA. Cell extracts were incubated with anti-TAP beads and analysed by immunoblotting. **F)** FACS analysis of the experiment in **E)**. **G)** Cells carrying several different N-terminally tagged truncations of *SEN1* under the *GAL1* promoter were grown to exponential phase in YPRAF, divided in two cultures and transferred to either fresh YPRAF or to YPGAL for 2 h. Protein extracts were analysed by immunoblotting with an anti-HA antibody. **H)** (Top) Cells carrying a temperature-sensitive allele *td-sen1-1* and different fragments of *SEN1* under the *GAL1-3HA* promoter were plated, according to the schematic presented, on YPD or YPGAL and incubated at either 24°C or 37°C. (Bottom) schematic of the plated strains. **I)** The interaction of Sen1 (2-931) with TAP-Mcm3 during S phase is specific. Experiments carrying an untagged or a TAP-tagged allele of *MCM3* were conducted as in Fig 1C. **J)** FACS samples from the experiment are shown.

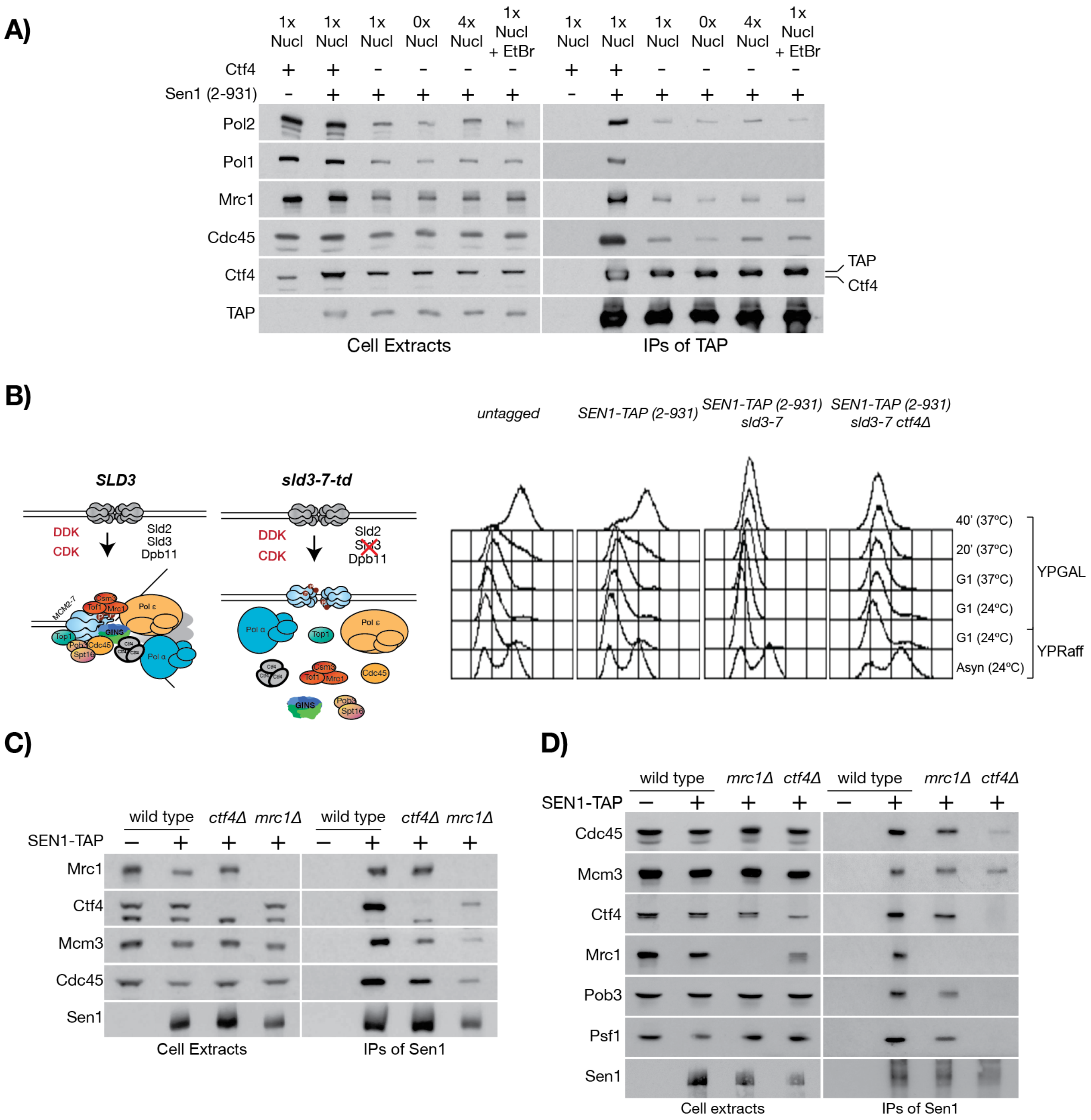

A)
1x Nucl
0x Nucl
4x Nucl
1x Nucl + EtBr
Ctf4
Sen1 (2-931)
Pol2
Pol1
Mrc1
Cdc45
Ctf4
TAP
TAP
Ctf4
Cell Extracts
IPs of TAP
B)
SLD3
sld3-7-td
DDK
CDK
Sld2
Sld3
Dpb11
Pol ε
Pol α
Top1
Cdc45
GINS
Csm3
Tof1
Mrc1
Pob3
Spt16
untagged
SEN1-TAP (2-931)
SEN1-TAP (2-931) sld3-7
SEN1-TAP (2-931) sld3-7 ctf4Δ
40' (37ºC)
20' (37ºC)
G1 (37ºC)
G1 (24ºC)
G1 (24ºC)
Asyn (24ºC)
YPGAL
YPRaff
C)
wild type
ctf4Δ
mrc1Δ
SEN1-TAP
Mrc1
Ctf4
Mcm3
Cdc45
Sen1
Cell Extracts
IPs of Sen1
D)
wild type
mrc1Δ
ctf4Δ
SEN1-TAP
Cdc45
Mcm3
Ctf4
Mrc1
Pob3
Psf1
Sen1
Cell extracts
IPs of Sen1

Figure S2. **Mrc1 and Ctf4 mediate Sen1 binding to the replisome** (related to Fig 2). **A)** Cells carrying the *GAL1-TAP-SEN1 (2- 931)* construct or the empty control were grown in YPGAL, arrested in G1 phase using α-factor and released in S phase for 20 min at 30°C. The samples were then used for IPs using TAP beads and treated with the indicated amount of nuclease or ethidium bromide (50 µg/ml). Ctf4 and TAP-Sen1 (2-931) have similar sizes and run closely in gel electrophoresis. **B)** (Left) Schematic representation of the system used in the experiment shown in Fig 2D; (Right) FACS profile of the experiment conducted. Cells were grown in YPRAF at 24°C, arrested in G1 and either harvested, or resuspended in YPGAL at 24°C for 35 min to induce the expression of Sen1 (2-931) and Ubr1, shifted to 37°C for 1 h to inactivate/degrade td-Sld3-7 and then released in S phase for 20 min at 37°C. **C)** Wild type, *ctf4*Δ and *mrc1*Δ cells, carrying a TAP-tagged or untagged allele of *SEN1*, were arrested in G1 and synchronously released in S phase for 30 min at 24˚C. Cell extracts were incubated with anti-TAP beads and the immunoprecipitated material was analysed by immunoblotting. **D)** Immunoblotting analysis of cell extracts and IP material from anti-TAP beads. The experiment was conducted an in **C)**, except cultures were incubated with formaldehyde before collection.

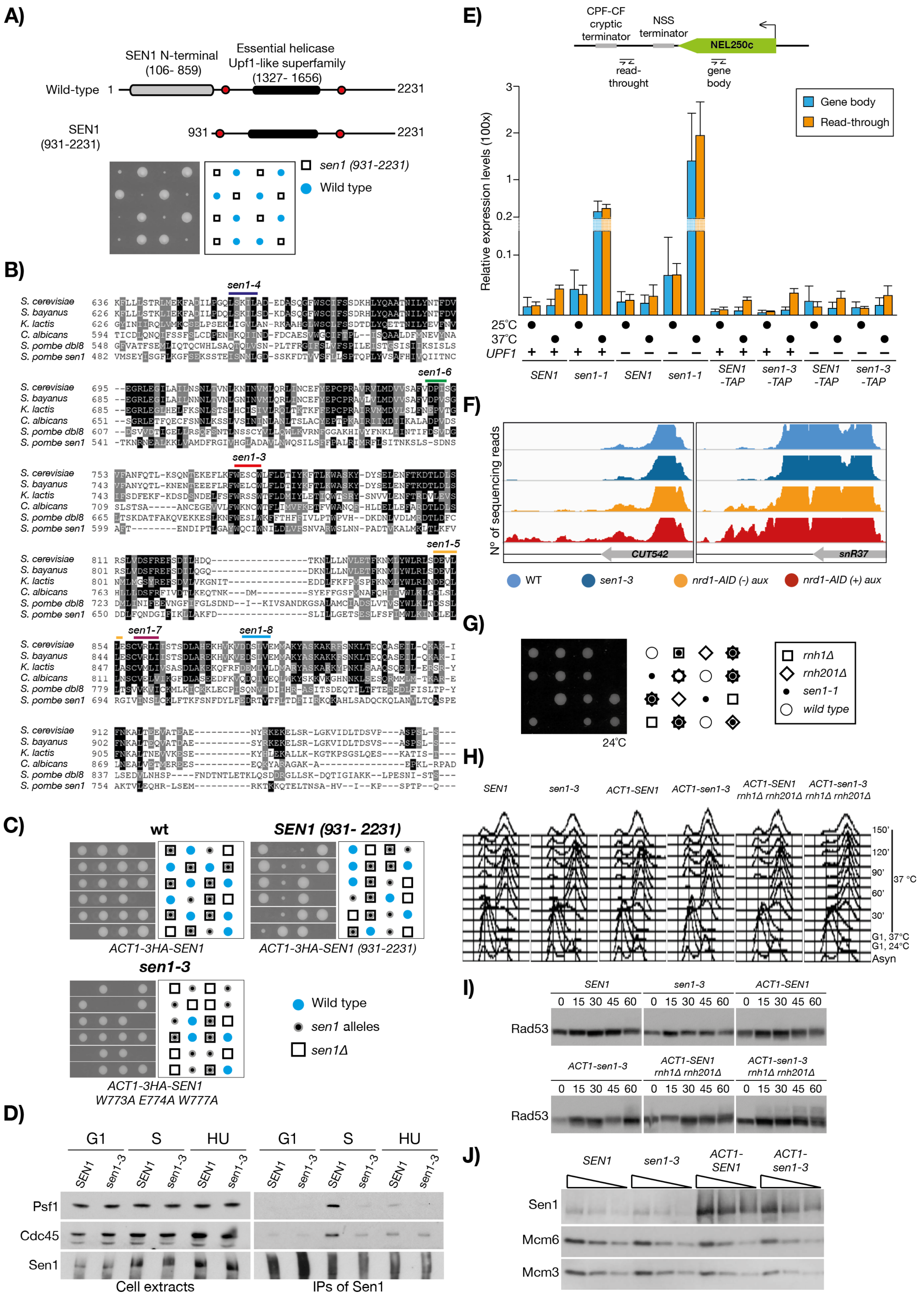
A)
SEN1 N-terminal
(106- 859)
Essential helicase
Upf1-like superfamily
(1327- 1656)
Wild-type 1
2231
SEN1
(931-2231)
931
2231
sen1 (931-2231)
Wild type
B)
sen1-4
sen1-6
sen1-3
sen1-5
sen1-7
sen1-8
S. cerevisiae
S. bayanus
K. lactis
C. albicans
S. pombe dbl8
S. pombe sen1
C)
wt
SEN1 (931- 2231)
ACT1-3HA-SEN1
ACT1-3HA-SEN1 (931-2231)
sen1-3
ACT1-3HA-SEN1
W773A E774A W777A
Wild type
sen1 alleles
sen1Δ
D)
G1
S
HU
SEN1
sen1-3
Psf1
Cdc45
Sen1
Cell extracts
IPs of Sen1
E)
CPF-CF
cryptic
terminator
NSS
terminator
NEL250c
read-
throught
gene
body
Relative expression levels (100x)
Gene body
Read-through
25˚C
37˚C
UPF1
SEN1
sen1-1
SEN1
-TAP
sen1-3
-TAP
F)
N° of sequencing reads
CUT542
snR37
WT
sen1-3
nrd1-AID (-) aux
nrd1-AID (+) aux
G)
24˚C
rnh1Δ
rnh201Δ
sen1-1
wild type
H)
SEN1
sen1-3
ACT1-SEN1
ACT1-sen1-3
ACT1-SEN1
rnh1Δ rnh201Δ
ACT1-sen1-3
rnh1Δ rnh201Δ
150'
120'
90'
60'
30'
37 °C
G1, 37°C
G1, 24°C
Asyn
I)
SEN1
sen1-3
ACT1-SEN1
0 15 30 45 60
Rad53
ACT1-sen1-3
ACT1-SEN1
rnh1Δ rnh201Δ
ACT1-sen1-3
rnh1Δ rnh201Δ
J)
SEN1
sen1-3
ACT1-
SEN1
ACT1-
sen1-3
Sen1
Mcm6
Mcm3

Figure S3. The N-terminal of Sen1 is important for cell growth and is conserved in yeasts (related to Fig 3 and 4)**. A)** Tetrad analysis of a diploid yeast strain carrying the *SEN1/sen1 (1-931Δ)* alleles. Plates were imaged after 5 days of growth on YPD at 24°C. **B)** Alignment of *Saccharomyces cerevisiae* Sen1 domain interacting with the replisome (636-931) with its orthologues from *Saccharomyces bayanus*, *Kluyveromyces lactis*, *Candida albicans* and *Schizosaccharomyces pombe dbl8* and *sen1*. The mutations used in the screen were selected to mutate conserved amino acids predicted to be on the surface (www.predictprotein.org). **C)** Analysis of the *ACT1-3HA-SEN1* mutants. Tetrad analyses were conducted on diploids yeast strains carrying *SEN1/sen1*Δ, and ectopically integrated *ACT1-3HA-SEN1* alleles at the *leu2-3,112* locus. Plates were imaged after 3 days of growth on YPDA at 24°C. **D)** Sen1-3 show greatly reduced interaction with RFs. Wild type and *sen1-3* cells were arrested in G1 and synchronously released for 30 min in fresh medium (S) or for 90 min in medium containing 0.2 M HU. At the indicated times, cultures were treated with formaldehyde before being collected. The cross-linked cell extracts and the immunoprecipitated material from anti-TAP beads were analysed by immunoblotting**. E)** (Top) Schematic representation of the gene analysed and the probes used to assess defects in transcription termination; (Bottom) RT-qPCR analysis of RNAs derived from the indicated strains. *NEL025c* is a non-coding region as described in (Wyers *et al.*, 2005). Cells were grown to exponential phase and incubated for 3 h at the indicated temperature before being collected. The signal is presented as the expression level relative to the housekeeping gene *ACT1* (triplicate biological repeats). **F)** Snapshots illustrating RNA Pol II density detected by CRAC on two NNS complex targets (one CUT and one snoRNA) in the indicated strains. An *nrd1-AID* strain grown in the presence or absence of auxin is included as a control for transcription termination (dataset from (Candelli *et al.*, 2018)). **G)** *sen1-1* is lethal in the absence of *RNH201* and *RNH1*. Examples are shown of tetrad analyses conducted from yeast diploids strains with the *SEN1/sen1-1 RNH1/rnh1*Δ and *RNH201/rnh201*Δ genotype. Plates were imaged after 4 days of growth on YPDA at 24°C. **H)** FACS analysis of the cell cycle progression in cells *SEN1*, *sen1-3, ACT1-SEN1, ACT1-sen1-3, ACT1-SEN1 rnh1*Δ *rnh201*Δ and *ACT1-sen1-3 rnh1*Δ *rnh201*Δ. Cells were grown to the exponential phase at 24°C, arrested in G1, shifted to 37°C for 1 hour in G1, and released in S phase at 37°C. The samples were collected at the indicated time points. **I)** *ACT1-sen1-3 rnh1*Δ *rnh201*Δ cells show activation of Rad53 during DNA replication at 37°C. Western blot analysis of samples taken from the experiment shown in **H). J)** Analysis of the protein levels of the alleles *SEN1*, *sen1-3*, *ACT1-SEN1* and *ACT1-sen1-3*, all carrying a 3HA tag, and loading controls Mcm3 and Mcm6.

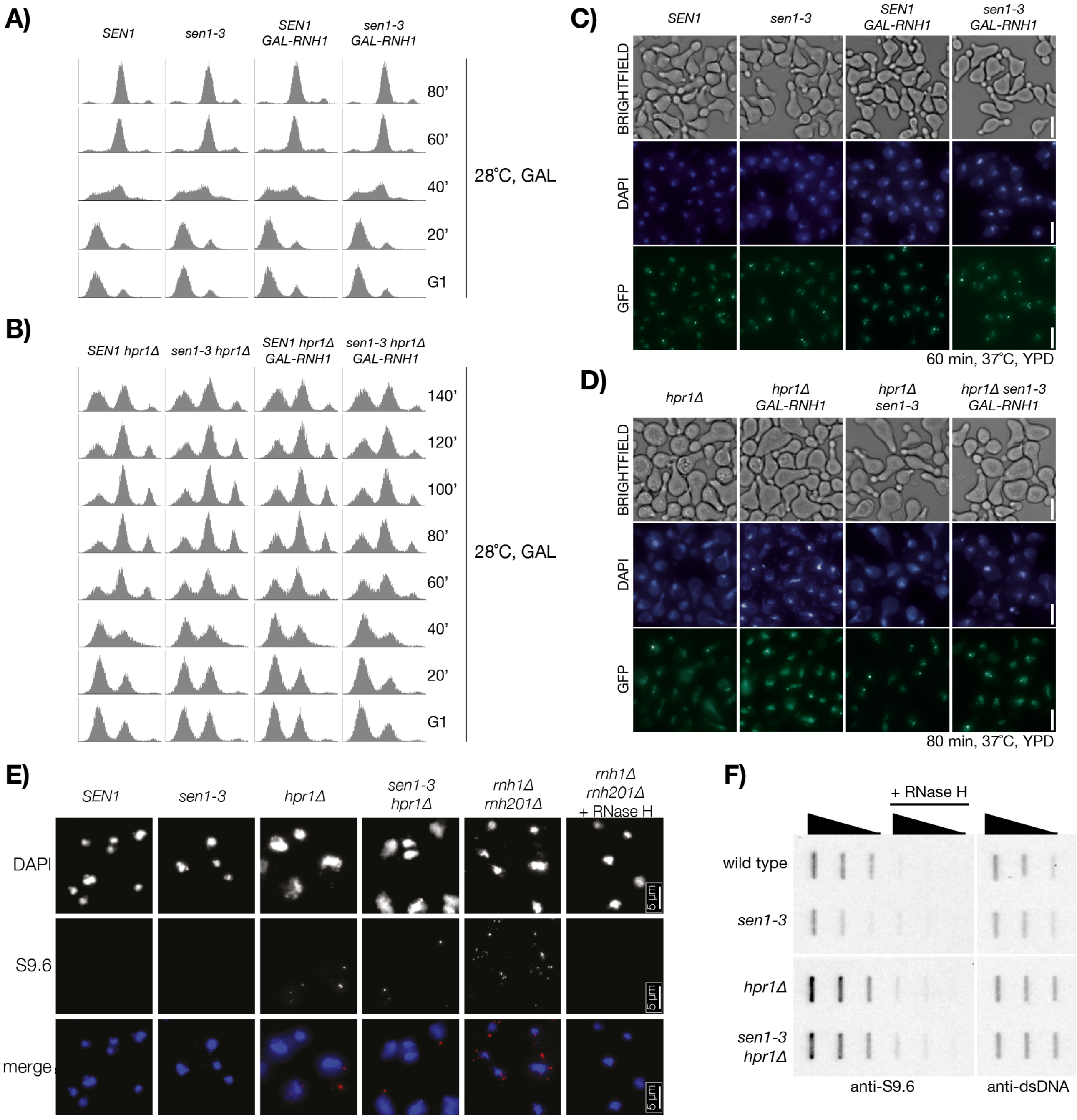
A)
SEN1
sen1-3
SEN1 GAL-RNH1
sen1-3 GAL-RNH1
80'
60'
40'
20'
G1
28°C, GAL
B)
SEN1 hpr1Δ
sen1-3 hpr1Δ
SEN1 hpr1Δ GAL-RNH1
sen1-3 hpr1Δ GAL-RNH1
140'
120'
100'
80'
60'
40'
20'
G1
28°C, GAL
C)
SEN1
sen1-3
SEN1 GAL-RNH1
sen1-3 GAL-RNH1
BRIGHTFIELD
DAPI
GFP
60 min, 37°C, YPD
D)
hpr1Δ
hpr1Δ GAL-RNH1
hpr1Δ sen1-3
hpr1Δ sen1-3 GAL-RNH1
BRIGHTFIELD
DAPI
GFP
80 min, 37°C, YPD
E)
SEN1
sen1-3
hpr1Δ
sen1-3 hpr1Δ
rnh1Δ rnh201Δ
rnh1Δ rnh201Δ + RNase H
DAPI
S9.6
merge
5 µm
F)
+ RNase H
wild type
sen1-3
hpr1Δ
sen1-3 hpr1Δ
anti-S9.6
anti-dsDNA

Figure S4. Analysis of the recombination and DNA:RNA hybrids in *hpr1*Δ and *hpr1*Δ *sen1-3* (related to Fig 4**). A-D)** FACS analysis of the DNA replication dynamics and examples of the microscopy data of the experiments shown in Fig 4F are shown. Cells were grown to exponential phase in YPRAF at 28°C, arrested in G1, resuspended in YPGAL for 1 h and synchronously released in S phase in YPGAL (triplicate biological repeats). Scale bar = 5 µm **E)** Examples of the immunohistochemistry analysis of DNA:RNA shown in Fig 4G (triplicate biological repeats). Scale bar = 5 µm **F)** Analysis of R-loops *ex vivo***.** Cells were grown to exponential phase, arrested in G1 and then synchronously released in S phase for 30 min at 24°C. DNA:RNA hybrids double-stranded DNA were recovered in nuclease-free water and 1, 0.5 and 0.25 µg/µl dilutions of nucleic acid samples were prepared. The samples were then either treated with a commercially-sourced RNase H (or mock-treated), transferred onto nylon membrane and probed against using either the S9.6 antibody (that recognise R-loops) or an anti-dsDNA antibody.

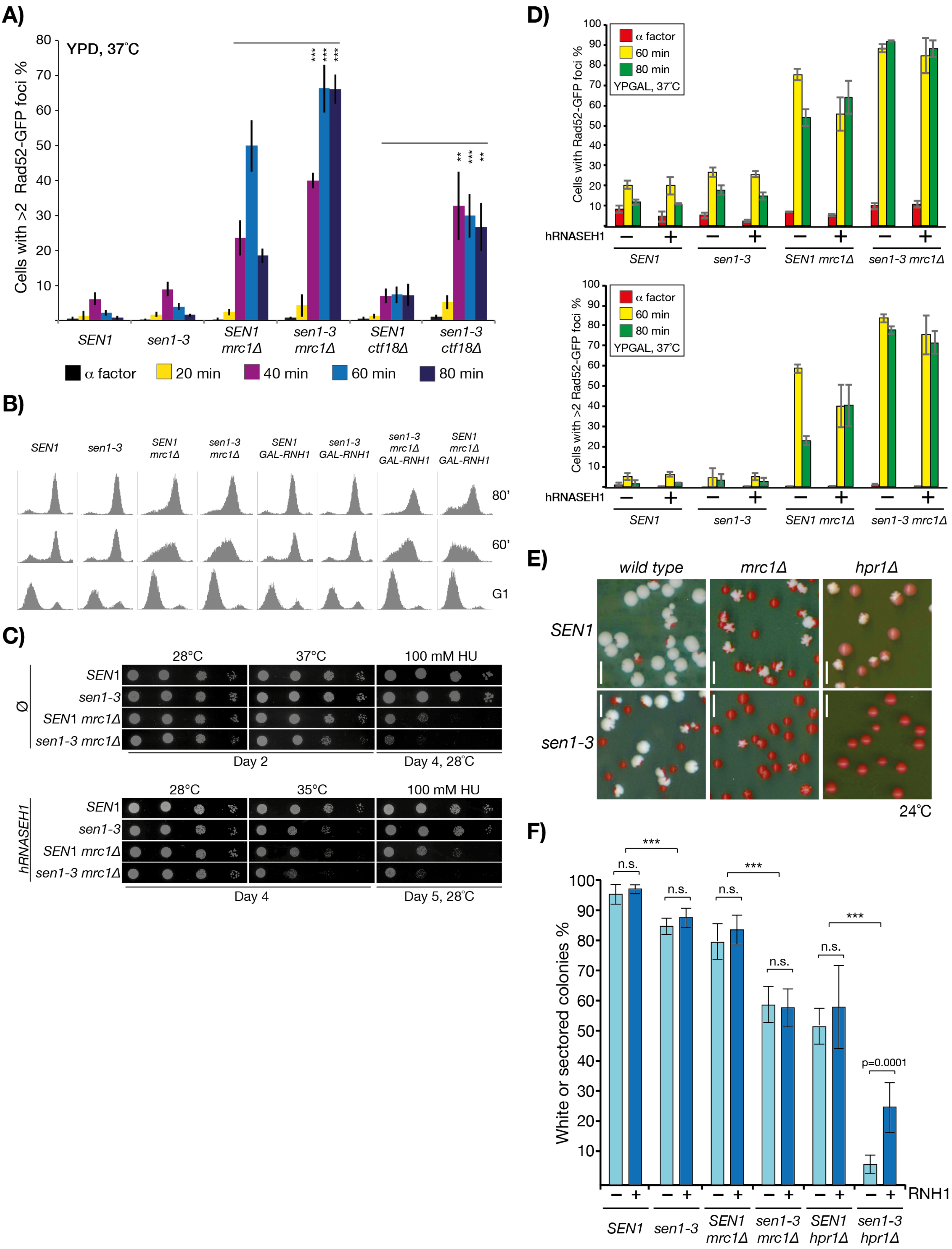

A)
YPD, 37°C
Cells with >2 Rad52-GFP foci %
SEN1
sen1-3
SEN1 mrc1Δ
sen1-3 mrc1Δ
SEN1 ctf18Δ
sen1-3 ctf18Δ
α factor
20 min
40 min
60 min
80 min
B)
SEN1
sen1-3
SEN1 mrc1Δ
sen1-3 mrc1Δ
SEN1 GAL-RNH1
sen1-3 GAL-RNH1
sen1-3 mrc1Δ GAL-RNH1
SEN1 mrc1Δ GAL-RNH1
80'
60'
G1
C)
28°C
37°C
100 mM HU
Ø
SEN1
sen1-3
SEN1 mrc1Δ
sen1-3 mrc1Δ
Day 2
Day 4, 28°C
28°C
35°C
100 mM HU
hRNASEH1
Day 4
Day 5, 28°C
D)
Cells with >2 Rad52-GFP foci %
α factor
60 min
80 min
YPGAL, 37°C
hRNASEH1
SEN1
sen1-3
SEN1 mrc1Δ
sen1-3 mrc1Δ
E)
wild type
mrc1Δ
hpr1Δ
SEN1
sen1-3
24°C
F)
White or sectored colonies %
n.s.
***
p=0.0001
RNH1
SEN1
sen1-3
SEN1 mrc1Δ
sen1-3 mrc1Δ
SEN1 hpr1Δ
sen1-3 hpr1Δ

Figure S5. Overexpression of *RNH1* does not suppress the defects in *mrc1*Δ *sen1-3* (related to Fig 5**)**. **A)** Samples from experiments show in Fig 5D were scored for the presence of two or more foci per cell. ** $p<0.05$, *** $p<0.01$). **B)** FACS analysis of the experiment shown in Fig 5G. **C)** *hRNASEH1* overexpression does not suppress the defects observed in *sen1-3 mrc1*Δ cells. *hRNASEH1* was overexpressed from a 2 micron multicopy plasmid under the strong *GPD* ^{TDH3} promoter. *SEN1*, *sen1-3*, *mrc1*Δ and *mrc1*Δ *sen1-3* cells were transformed with an empty or *GPD*^{TDH3}-*hRNASEH1* plasmid. Eight independent clones were pooled together and used for dilution spotting in medium lacking histidine, so to maintain the selective pressure for the plasmid. The cells carrying *GDP-hRNH1* grew more slowly and scans of their growth were taken at later times (Bottom panel). Serial dilution spotting (1:10) of the indicated strains is shown. **D)** *hRNASEH1* overexpression does not suppress the increase in recombination in *sen1-3 mrc1*Δ. Cell cultures were grown overnight at 24°C in medium lacking histidine to the exponential phase. Cells were diluted, resuspended in YPD, and left to grow for the length of one cell cycle. Cells were arrested in G1, shifted to 37°C for 1 h still with α-factor, and released in S phase at 37°C. Cells were taken at the indicated times, fixed, and analysed (triplicate biological repeats). **E)** Examples of the plasmid loss phenotype observed in the strains shown in Fig 5I (plasmid with 1 origin). Scale bar: 5 mm. **F)** The indicated strains, either carrying the *GAL1-RNH1* construct integrated at *leu2-3,112* or not, were transformed with the pRS315-*ADE2* plasmid. The experiment was performed as in Fig 5I, except that all media used contained galactose. n.s. = not significant, *** $p<0.001$.
